# Polymersomes with splenic avidity target red pulp myeloid cells for cancer immunotherapy

**DOI:** 10.1038/s41565-024-01727-w

**Published:** 2024-07-31

**Authors:** Annelies C. Wauters, Jari F. Scheerstra, Mandy M. T. van Leent, Abraham J. P. Teunissen, Bram Priem, Thijs J. Beldman, Nils Rother, Raphaël Duivenvoorden, Geoffrey Prévot, Jazz Munitz, Yohana C. Toner, Jeroen Deckers, Yuri van Elsas, Patricia Mora-Raimundo, Gal Chen, Sheqouia A. Nauta, Anna Vera D. Verschuur, Arjan W. Griffioen, David P. Schrijver, Tom Anbergen, Yudong Li, Hanglong Wu, Alexander F. Mason, Marleen H. M. E. van Stevendaal, Ewelina Kluza, Richard A. J. Post, Leo A. B. Joosten, Mihai G. Netea, Claudia Calcagno, Zahi A. Fayad, Roy van der Meel, Avi Schroeder, Loai K. E. A. Abdelmohsen, Willem J. M. Mulder, Jan C. M. van Hest

**Affiliations:** 1 Bio-Organic Chemistry, Institute for Complex Molecular Systems, Eindhoven University of Technology, Eindhoven, the Netherlands; 2 BioMedical Engineering and Imaging Institute, Icahn School of Medicine at Mount Sinai, New York, NY USA; 3 Cardiovascular Research Institute, Icahn School of Medicine at Mount Sinai, New York, NY USA; 4 Icahn Genomics Institute, Icahn School of Medicine at Mount Sinai, New York, NY USA; 5 Experimental Vascular Biology, Department of Medical Biochemistry, Amsterdam Cardiovascular Sciences (ACS), Amsterdam University Medical Center, Amsterdam, the Netherlands; 6 Department of Medical Oncology (NA Angiogenesis Laboratory), Amsterdam University Medical Center, Cancer Center Amsterdam, Amsterdam, the Netherlands; 7 Department of Internal Medicine, Radboud University Medical Center, Nijmegen, the Netherlands; 8 Department of Nephrology, Radboud University Medical Center, Nijmegen, the Netherlands; 9 The Luis Family Laboratory for Targeted Drug Delivery and Personalized Medicine Technologies, Department of Chemical Engineering, Technion, Haifa, Israel; 10 Laboratory of Chemical Biology, Department of Biomedical Engineering and Institute for Complex Molecular Systems, Eindhoven University of Technology, Eindhoven, the Netherlands; 11 Department of Mathematics and Computer Science, Institute of Complex Molecular Systems, Eindhoven University of Technology, Eindhoven, the Netherlands; 12 Department of Medical Genetics, Iuliu Hatieganu University of Medicine and Pharmacy, Cluj-Napoca, Romania; 13 Department for Genomics and Immunoregulation, Life and Medical Sciences Institute, University of Bonn, Bonn, Germany

**Keywords:** Nanotechnology in cancer, Drug delivery

## Abstract

Regulating innate immunity is an emerging approach to improve cancer immunotherapy. Such regulation requires engaging myeloid cells by delivering immunomodulatory compounds to hematopoietic organs, including the spleen. Here we present a polymersome-based nanocarrier with splenic avidity and propensity for red pulp myeloid cell uptake. We characterized the in vivo behaviour of four chemically identical yet topologically different polymersomes by in vivo positron emission tomography imaging and innovative flow and mass cytometry techniques. Upon intravenous administration, relatively large and spherical polymersomes accumulated rapidly in the spleen and efficiently targeted myeloid cells in the splenic red pulp. When loaded with β-glucan, intravenously administered polymersomes significantly reduced tumour growth in a mouse melanoma model. We initiated our nanotherapeutic’s clinical translation with a biodistribution study in non-human primates, which revealed that the platform’s splenic avidity is preserved across species.

## Abstract (web summary)

Delivering immunomodulatory compounds to myeloid cells can activate innate immunity for cancer immunotherapy. Here the authors design a polymersome-based nanocarrier for delivering β-glucan to red pulp myeloid cells in the spleen and show that their strategy achieves tumour growth reduction in a melanoma model.

## Main

Immunotherapy using checkpoint inhibitor drugs to activate antitumour T cells has rapidly matured into a strategy for treating various cancer types^1^. However, therapeutic efficacy and applicability remain limited as most patients do not respond well to checkpoint inhibition, and life-threatening toxicities can occur^2^. Systemically released immunosuppressive myeloid cells contribute to the impairment of checkpoint blockade therapy, by causing accumulation of tumour-associated macrophages and myeloid-derived suppressor cells in the tumour microenvironment that promote tumour progression^3–5^. Myeloid cell-directed strategies are therefore increasingly being considered to turn a ‘cold’ unresponsive tumour into a ‘hot’ immunogenic site^6–9^.

Myeloid cells, part of the innate immune system, include neutrophils, monocytes, macrophages and dendritic cells. These cells are generated by myelopoiesis in the bone marrow medulla and reside in many other tissues, including the lymph nodes and spleen^3^. Although underexplored, the spleen has emerged as the major site for extramedullary myelopoiesis and acts as a monocyte reservoir^10–12^. Emerging evidence demonstrates that tumours alter splenic myelopoiesis and replenish immunosuppressive myeloid cells from the splenic reservoir^13–16^. Therefore, targeting splenic myeloid cells to reverse systemic and local immunosuppression boosts and diversifies cancer immunotherapy^17^.

Many biomolecules can stimulate myeloid cells in vitro^18^. However, the effects of these compounds are often not recapitulated in vivo owing to their poor bioavailability to myeloid cells. A specific example are β-glucans, a class of polysaccharides capable of effectively inducing trained immunity in monocytes in vitro^19–22^. Trained immunity is a metabolically and epigenetically regulated functional innate immune state characterized by increased cellular responsiveness that can be leveraged to reduce tumour growth^8,23^. In this regard, β-glucans have recently been shown to prophylactically and therapeutically enhance resistance against cancer^24–26^. However, the full potential of these polysaccharides in cancer management has yet to be realized. Optimally exploiting β-glucans for innate immune regulation in vivo requires their direct engagement with the dectin-1 receptor expressed by myeloid cells^27^. This can be achieved by delivering β-glucans to primary or secondary lymphoid organs, including the spleen^10,12^.

Nanomedicine is a promising and emerging approach for innate immunotherapy^28^, by utilizing nanocarriers^29,30^ that can effectively target myeloid cells in the bone marrow^8,31^, lymph nodes^32,33^ or spleen^34^. Traditionally, the nanomedicine field primarily focused on prolonging nanocarrier circulation to take advantage of the enhanced permeability and retention effect to accumulate chemotherapeutic agents in tumours more safely. This was typically achieved by surface-modifying the nanocarrier with hydrophilic polymers, reducing interactions with phagocytes and preventing premature clearance. However, it is becoming increasingly clear that nanocarriers’ inherent interaction with phagocytes makes them exceptionally suitable for innate immunotherapy^28,32,35^.

Developing nanomedicine-based immunotherapies for targeting specific tissues and cell types requires systematically optimizing the administration route and physicochemical features of the nanocarrier, such as size and shape. Furthermore, to facilitate clinical translation, it is critical to ensure that nanocarrier properties observed in animal studies are preserved in patients. This requires thorough in vivo studies of nanocarrier behaviour in both small lab animals, for example, mice, and clinically more relevant animals, such as non-human primates (NHPs)^36,37^.

**Fig. 1:**
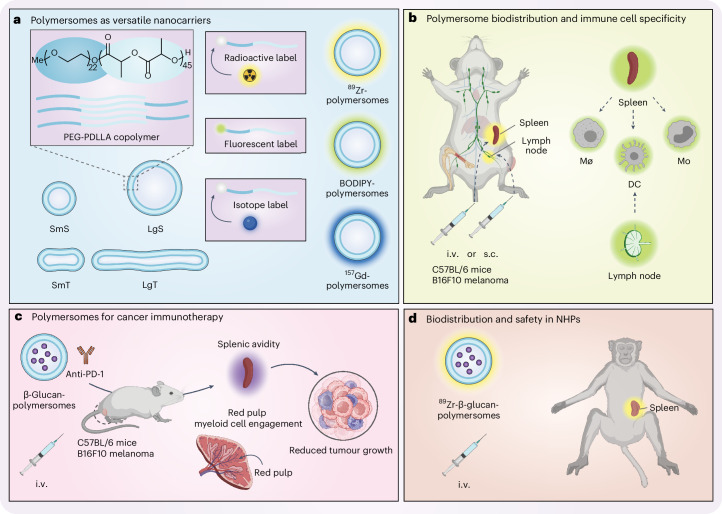
Schematic overview of the study. **a**, Polymersomes consist of biodegradable PEG-PDLLA block copolymers assembled into bilayered vesicles. Polymersome topology can be precisely controlled to form spheres and tubes of different sizes (SmS and LgS denote small and large spheres and SmT and LgT denote small and large tubes, respectively). Furthermore, polymersomes are labelled with radionuclides (^89^Zr), fluorescent dyes (BODIPY) or stable isotopes (^157^Gd). **b**, Polymersome in vivo biodistribution was assessed by PET/CT imaging in B16F10 melanoma-bearing C57BL/6 mice upon polymersome administration intravenously (i.v.) via lateral tail vein injection or subcutaneously (s.c.) via footpad injection. Using flow and mass cytometry, we studied polymersome uptake by specific immune cells, including the different splenic compartments, that is, white pulp, red pulp and the marginal zone. **c**, We loaded polymersomes with β-glucan, applied either as a monotherapy or in combination with anti-PD-1 checkpoint inhibition. **d**, To study the clinical potential of our nanotherapeutic, we evaluated the biodistribution and biocompatibility of the large spherical β-glucan-polymersomes in NHPs by PET/CT imaging. DC, dendritic cell; MΦ, macrophage; Mo, monocyte. Schematic illustrations of mice, spleens, lymph nodes, syringes, cells, antibodies and NHPs were created with BioRender.com.

In this Article, we report on the in vivo behaviour of polymeric vesicles, or polymersomes^38^. Our nanosized polymersomes are composed of biodegradable block copolymers and exhibit structural and chemical versatility^39,40^. Furthermore, the polymersomes’ bilayered structure allows their aqueous core to be loaded with water-soluble cargo^40^. Although numerous reports describe polymersomes’ topological aspects in nanomedicine applications in vitro, their in vivo behaviour remains poorly understood^41–44^. We developed a library of four polymersomes with identical composition but varying topology, namely small spheres (SmS), small tubes (SmT), large spheres (LgS) and large tubes (LgT) (Fig. 1a). Following subcutaneous or intravenous administration, we extensively profiled the polymersomes’ pharmacokinetics, biodistribution and immune cell engagement in B16F10 melanoma-bearing C57BL/6 mice (Fig. 1b). Using a combination of in vivo positron emission tomography (PET) imaging and multi-organ flow and mass cytometry protocols, we selected the optimal polymersome topology and administration route for rapid splenic accumulation and efficient red pulp myeloid cell uptake. As a proof of concept, we incorporated a water-soluble low-molecular-weight β-glucan (laminarin) into this lead formulation to yield β-glucan-polymersomes. We investigated β-glucan-loaded polymersomes as a monotherapy and in combination with anti-PD-1 checkpoint inhibition in the B16F10 mouse melanoma model (Fig. 1c). Finally, we gauged the translational potential of β-glucan-polymersomes using biodistribution and biocompatibility studies in NHPs (Fig. 1d).

## Establishing and labelling a polymersome library

**Fig. 2:**
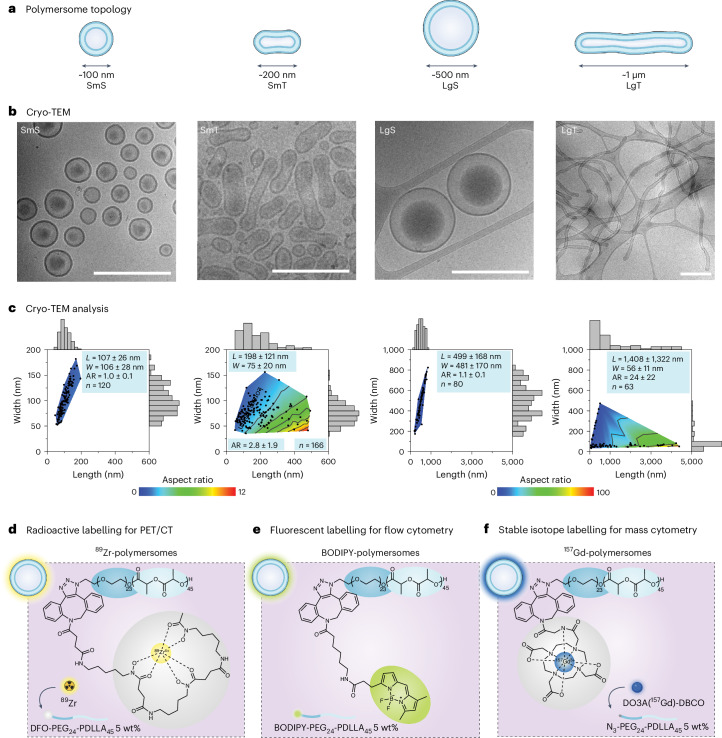
Characterizing and labelling polymersomes. **a**, Schematic representation of the four different polymersome topologies used in this study. **b**, Representative cryo-TEM micrographs of the four BODIPY-labelled polymersome topologies in their respective dialysis fluids, that is, SmS and LgS, were measured in Milli-Q water and SmT and LgT in 50 mM NaCl. Scale bars, 500 nm. Similar results for polymersome topologies were obtained in three independent experiments. **c**, Polymersome dimensions determined using the cryo-TEM micrographs. The heatmap indicates the corresponding polymersome aspect ratios. **d**, Schematic representation of the polymersome radiolabelling strategy. DFO-functionalized polymersomes were formulated by incorporating 5 wt% DFO-PEG_24_-PDLLA_45_ followed by incubation with radioactive ^89^Zr to yield ^89^Zr-polymersomes. **e**, Schematic representation of the polymersome fluorescent labelling strategy. BODIPY-polymersomes were formulated by incorporating 5 wt% BODIPY-PEG_24_-PDLLA_45_. **f**, Schematic representation of the polymersome stable isotype labelling strategy. N_3_-functionalized polymersomes were formulated by incorporating 5 wt% N_3_-PEG_24_-PDLLA_45_. These polymersomes were subsequently incubated with stable ^157^Gd complexed in DO3A-DBCO to yield ^157^Gd-polymersomes. All data are represented as mean ± s.d.

To systematically assess the effects of nanocarrier topology on biodistribution and immune cell affinity, we constructed four polymersomes based on our previously reported poly(ethylene glycol)-block-poly(d,l-lactide) (PEG-PDLLA) platform^39,40^. We precisely regulated polymersome topology by consecutive self-assembly, extrusion and osmotically induced shape transformation processes, resulting in spherical or tubular nanostructures with well-defined sizes and aspect ratios. Specifically, we established a small library of four polymersomes, namely SmS (ca. 100 nm diameter), SmT (ca. 200 × 80 nm length × width), LgS (ca. 500 nm diameter) and LgT (ca. 1 µm × 60 nm length × width) (Fig. 2a). Supplementary Figs. 1–3 and Supplementary Fig. 4 describe polymer synthesis and polymersome formulation, respectively. Cryogenic transmission electron microscopy (cryo-TEM) imaging corroborated polymersome topology (Fig. 2b and Supplementary Fig. 5). Aspect ratios (AR; defined as polymersome length/width) were obtained by analysing cryo-TEM micrographs and were 1.0 for SmS, 1.1 for LgS, 2.8 for SmT and 24 for LgT (Fig. 2c). Dynamic light scattering (DLS), nanoparticle tracking analysis (NTA) and asymmetric flow field-flow fractionation coupled with multi-angle static and quasi-elastic light scattering (AF4-MALS-QELS) corroborated the polymersome topologies (Supplementary Table 1 and Supplementary Fig. 6). A combination of DLS and cryo-TEM verified that the polymersomes’ tubular morphology is stable under physiological conditions (Supplementary Fig. 7).

To study the polymersomes’ in vivo behaviour, we incorporated 5 wt% of DFO-PEG_24_-PDLLA_45_ to enable their labelling with the radioisotope zirconium-89 (^89^Zr, *t*_1/2_ ≈ 78 h; Fig. 2d). ^89^Zr-polymersomes can be monitored by highly sensitive quantitative in vivo PET imaging and ex vivo gamma counting^45,46^. To investigate the polymersome immune cell specificity by flow and mass cytometry techniques, we incorporated the fluorescent polymer BODIPY-PEG_24_-PDLLA_45_ into the polymersomes (Fig. 2e) or conjugated them to DO3A by click chemistry. The latter allows labelling of the polymersomes with the stable isotope gadolinium-157 (^157^Gd; Fig. 2f) and therefore facilitates their tracking by mass cytometry.

## Polymersome topology dictates splenic myeloid cell avidity

**Fig. 3:**
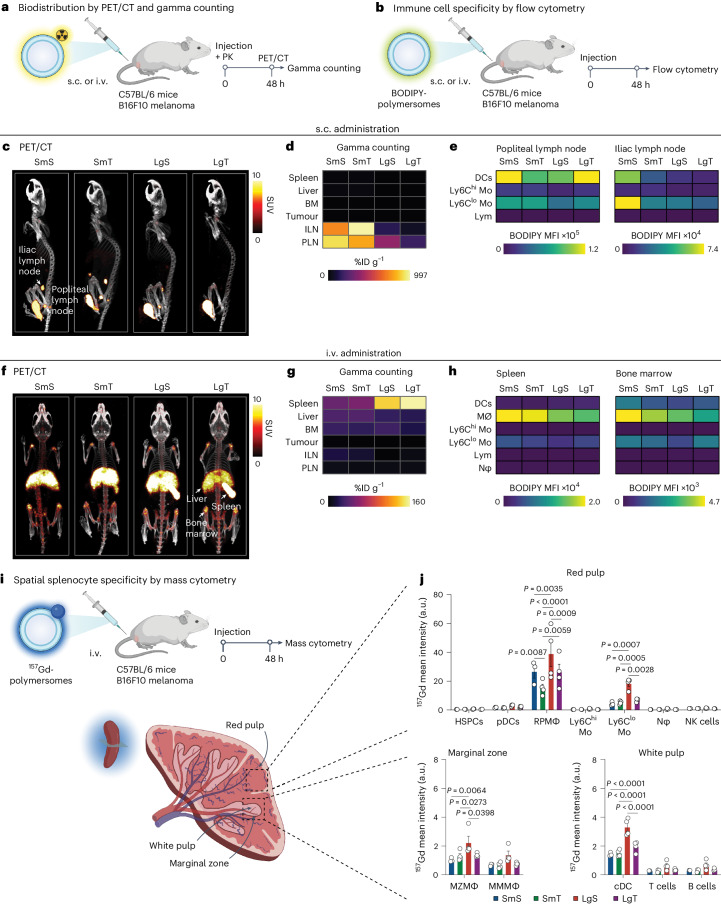
Polymersome biodistribution and immune cell specificity in the B16F10 melanoma mouse model. **a**,**b**, Schematic overview of ^89^Zr-polymersome biodistribution studies by PET/CT imaging (**a**) and BODIPY-polymersome immune cell specificity studies by flow cytometry (**b**). **c**, Representative whole-body PET images taken 48 h after subcutaneous administration of ^89^Zr-polymersomes via footpad injection. **d**, Biodistribution of subcutaneously administered ^89^Zr-polymersomes, *n* = 4 per group. **e**, Uptake of subcutaneously administered BODIPY-polymersomes in specific immune cell types in the popliteal and iliac lymph nodes, *n* = 4 per group. **f**, Representative whole-body PET images taken 48 h after intravenous administration of ^89^Zr-polymersomes via lateral tail vein injection. **g**, Biodistribution of intravenously administered ^89^Zr-polymersomes, *n* = 4 per group. **h**, Uptake of intravenously administered BODIPY-polymersomes by immune cells in the spleen and bone marrow, *n* = 3 per group. Heatmaps of **d** and **g** show the average percentage injected dose per gram of tissue (%ID g^−1^) of the ^89^Zr-polymersomes per tissue as measured by ex vivo gamma counting. Heatmaps of **e** and **h** show the average BODIPY mean fluorescence intensity (MFI) per cell type. **i**, Schematic overview of ^157^Gd-polymersome splenocyte specificity study by mass cytometry. **j**, Uptake of intravenously administered ^157^Gd-polymersomes by red pulp, marginal zone or white pulp-resident splenocytes. The average ^157^Gd mean intensity for each cell type, *n* = 4 per group. BM, bone marrow; cDCs, classical DCs; HSPCs, hematopoietic stem and progenitor cells; ILN, iliac lymph node; Lym, lymphocytes; MZMΦφ, marginal zone macrophages; MMMΦ, marginal metallophilic macrophages; Nφ, neutrophils; NK cells, natural killer cells; PK, pharmacokinetics; PLN, popliteal lymph node; pDCs, plasmacytoid DCs; RPMΦ, red pulp macrophages. Data are presented as mean ± s.e.m. *P* values were calculated using a two-way ANOVA to compare polymersome topology per splenocyte subset. All *P* values are two-tailed and *P* < 0.05 is considered significant. Schematic illustrations of mice, syringes and spleens were created with BioRender.com. Source data

We evaluated our polymersome biodistribution (Fig. 3a) and immune cell specificity (Fig. 3b) in C57BL/6 mice inoculated subcutaneously with B16F10 melanoma tumours in their left flanks. ^89^Zr-polymersomes were administered in the animals’ left hind footpad to reach the lymphatics and lymph nodes near their tumours. In vivo PET/computed tomography (CT) imaging was performed two days after injection. We found that the SmS and SmT efficiently accumulated in the popliteal and iliac lymph nodes (Fig. 3c). These results were corroborated by ex vivo gamma counting, which showed 740% of injected dose per gram of tissue (%ID g^−1^) and 997 %ID g^−1^ of SmS and SmT in the iliac lymph nodes, respectively (Fig. 3d and Supplementary Fig. 8). By contrast, LgS and LgT showed much lower accumulation in the popliteal and iliac lymph nodes, as confirmed by gamma counts of only 140 %ID g^−1^ and 65 %ID g^−1^ in the iliac lymph nodes, respectively. Next, we used flow cytometry to evaluate the immune cell specificity of the polymersomes in these draining lymph nodes and found high myeloid cell avidity and low B cell and T cell uptake for all four BODIPY-polymersomes (Fig. 3e and Supplementary Fig. 9).

Next, we investigated the in vivo behaviour of ^89^Zr-polymersomes after intravenous administration. We observed blood half-lives of 10 min, 16 min, 30 min and 48 min for the SmS, SmT, LgS and LgT, respectively (Supplementary Fig. 8). In vivo PET imaging 48 h after intravenous injection revealed a drastically different polymersome biodistribution pattern compared with subcutaneous administration, with pronounced polymersome accumulation in the spleen, liver and bone marrow (Fig. 3f). The LgS and LgT accumulated in the spleen at 143 %ID g^−1^ and 161 %ID g^−1^, while their uptakes in the liver were 27 %ID g^−1^ and 23 %ID g^−1^, respectively (Fig. 3g and Supplementary Fig. 8). The SmS and SmT also accumulated in the spleen, but at much lower levels (48 %ID g^−1^ and 55 %ID g^−1^, respectively). Bone marrow uptake did not notably depend on polymersome size and ranged from 21 %ID g^−1^ (LgT) to 31 %ID g^−1^ (LgS). We again evaluated the polymersomes’ immune cell specificity by flow cytometry, focusing on the spleen and bone marrow. After intravenous administration of BODIPY-polymersomes, we observed a low BODIPY signal in lymphocytes and short-lived myeloid cells (neutrophils and Ly6C^hi^ monocytes), whereas macrophages showed the highest fluorescence intensity, indicating considerable polymersome uptake (Fig. 3h and Supplementary Figs. 10 and 11). We observed a similar cell specificity pattern for all four polymersomes in the spleen and bone marrow.

To enhance our understanding of the polymersome cellular distribution in the spleen, we used mass cytometry to investigate their uptake in resident cells of different splenic compartments, that is, the red pulp, marginal zone and white pulp (Fig. 3i). For all four ^157^Gd-polymersomes, we found substantial accumulation in red pulp macrophages and red pulp-resident Ly6C^lo^ monocytes (Fig. 3j and Supplementary Fig. 12). Red pulp macrophages showed significantly higher uptake of large polymersomes compared with their smaller counterparts (*P* = 0.0035 for spheres and *P* = 0.0059 for tubes; Fig. 3j). Moreover, tubes showed significantly lower accumulation in red pulp macrophages compared with spheres of similar size (*P* = 0.0087 for small and *P* = 0.0009 for large polymersomes; Fig. 3j). Compared with all other topologies, LgS showed higher accumulation in Ly6C^lo^ monocytes (*P* = 0.0007, *P* = 0.0005 and *P* = 0.0028 versus SmS, SmT and LgT respectively; Fig. 3j). While the LgS primarily accumulated in red pulp myeloid cells, their accumulation in marginal zone and white pulp myeloid cells was higher than the other topologies (Fig. 3j).

On the basis of the LgS high uptake in the spleen and favourable avidity for myeloid cells in the red pulp, we selected this nanocarrier and administration route for therapeutic studies.

## β-Glucan-polymersome immunotherapy reduces tumour growth

**Fig. 4:**
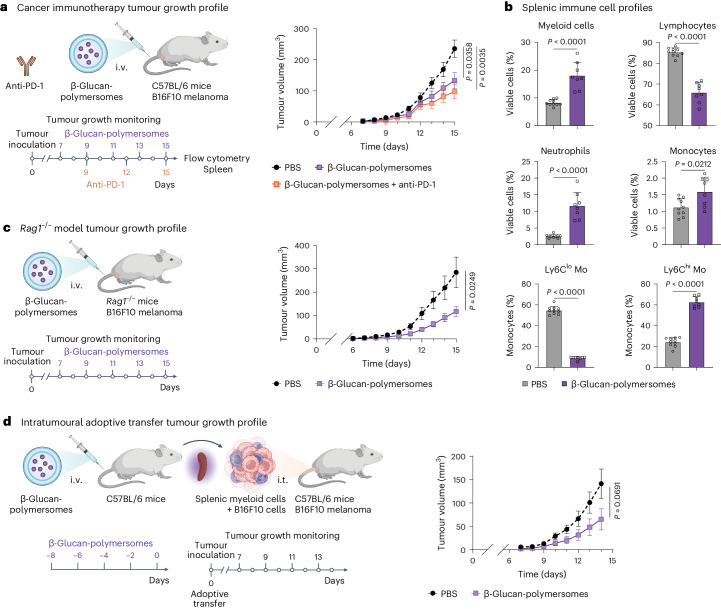
β-Glucan-loaded polymersomes modulate splenic myeloid cells and reduce tumour growth in the B16F10 melanoma mouse model. **a**, β-Glucan-polymersomes, as a monotherapy or combined with anti-PD-1 checkpoint inhibition, were administered intravenously to B16F10 melanoma-bearing C57BL/6 mice and tumour growth was monitored for 8 days. The tumour growth profiles show that β-glucan-polymersome immunotherapy effectively inhibited tumour growth as a monotherapy and in combination with anti-PD-1 checkpoint therapy, *n* = 10 per group. **b**, Relative abundance of splenic myeloid cells, lymphocytes, neutrophils, monocytes, Ly6C^lo^ monocytes and Ly6C^hi^ monocytes in B16F10 melanoma-bearing C57BL/6 mice treated with PBS and β-glucan-polymersomes. The abundance of splenic myeloid cells, including neutrophils and monocytes, was significantly increased in mice treated with β-glucan-polymersomes compared with PBS. The frequency of splenic Ly6C^lo^ monocytes was significantly lower, whereas splenic Ly6C^hi^ monocytes were significantly more abundant in mice treated with β-glucan-polymersomes, *n* = 8–10 per group. **c**, β-Glucan-polymersomes were administered intravenously to B16F10 melanoma-bearing *Rag1*^−^^/^^−^ mice and tumour growth was monitored for 8 days. Tumour growth profiling revealed that β-glucan-polymersome immunotherapy effectively inhibited tumour growth in lymphocyte-deficient *Rag1*^−/−^ mice, *n* = 10 per group. **d**, Healthy C57BL/6 mice were treated intravenously with β-glucan-polymersomes and splenic myeloid cells were isolated and co-inoculated with B16F10 melanoma cells. Tumour growth was monitored for 8 days. Results show that β-glucan-polymersome-treated myeloid cells inhibited tumour growth when co-inoculated with B16F10 melanoma cells, *n* = 10 per group. Tumour growth data are presented as mean ± s.e.m. and flow cytometry data are presented as mean ± s.d. *P* values were calculated using a repeated measures two-way ANOVA to compare tumour growth or an unpaired Mann–Whitney test to compare splenic immune cells. All *P* values are two-tailed and *P* < 0.05 is considered significant. i.t., intratumoural. Schematic illustrations of mice, spleens, syringes and cells were created with BioRender.com. Source data

As a proof-of-concept innate immunotherapy strategy, we incorporated laminarin in large, spherical polymersomes (Fig. 4a). Laminarin is a water-soluble low-molecular-weight β-glucan known to inhibit tumour growth^47^. Laminarin was encapsulated in the polymersome aqueous core during the formulation process and unincorporated β-glucan was removed by dialysis. After purification, β-glucan concentration was assessed using a validated colorimetric carbohydrate assay. β-Glucan concentration was determined to be 0.9 mg ml^−1^ (encapsulation efficiency ca. 1%) and block copolymer concentration was 40 mg ml^−1^ (Supplementary Fig. 13). See Supplementary Table 2 and Supplementary Fig. 13 for further β-glucan-polymersome characterization by DLS, NTA and cryo-TEM. Biodistribution studies of ^89^Zr-labelled β-glucan-polymersomes (^89^Zr-β-glucan-polymersomes) by positron emission tomography/computed tomography (PET/CT) imaging and gamma counting demonstrated that laminarin incorporation does not affect the in vivo behaviour of the platform (Supplementary Fig. 14).

Subsequently, we evaluated β-glucan-polymersomes’ therapeutic activity by treating B16F10 melanoma-bearing C57BL/6 mice. The B16F10 melanoma model, like clinical melanomas in general, is known for its immunogenicity and resistance to checkpoint inhibitor therapy^48^. In a previous study, we demonstrated that a nanobiologic platform engaging myeloid progenitors and inducing trained immunity in the bone marrow could sensitize B16F10 tumours to checkpoint inhibitor drugs^8^. Motivated by these results, we aimed to study β-glucan-polymersomes both as a monotherapy (intravenous injections of β-glucan-polymersomes every other day at 2.5 mg β-glucan per kg with a 5 mg kg^−1^ induction dose at day 7) and in combination with checkpoint inhibitor drugs (200 μg anti-PD-1 injected intraperitoneally at days 9, 12 and 15; Fig. 4a and Supplementary Fig. 15). β-Glucan-polymersomes (*P* = 0.0385) and the combination therapy (*P* = 0.0035) significantly reduced tumour growth in mice, compared with PBS treatment, without a strong synergistic effect (Fig. 4a). Neither free β-glucan, unloaded polymersomes, anti-PD-1 monotherapy nor prophylactic β-glucan-polymersome administration significantly changed tumour growth compared with the PBS control (Supplementary Fig. 15).

The high accumulation of β-glucan-polymersomes in the splenic myeloid cell compartment and relatively low tumour uptake enticed us to hypothesize that its antitumour effects stem predominantly from myeloid cell immunomodulation in the spleen. To investigate this, we performed flow cytometry on splenocytes from mice treated either with β-glucan-polymersomes or PBS control. We observed 2.2-fold higher myeloid cell abundance in β-glucan-polymersome-treated mice compared with mice treated with PBS control (*P* < 0.001), a change driven by both neutrophil and monocyte population expansions or influx (*P* < 0.001 and *P* = 0.0217, respectively; Fig. 4b and Supplementary Fig. 15). This expansion is further supported by an approximately 4-fold (*P* < 0.0001) increase in spleen weight that can be attributed to increased counts of myeloid cells, including neutrophils, monocytes but not lymphocytes (*P* < 0.01, *P* < 0.0001 and *P* < 0.05, respectively; Supplementary Fig. 15). β-Glucan-polymersome-treated mice had mainly pro-inflammatory, Ly6C^hi^ monocytes, whereas anti-inflammatory, Ly6C^lo^ monocytes were most abundant in the spleen of PBS-treated mice, as demonstrated by both cell frequencies and absolute cell counts (*P* < 0.0001 and *P* < 0.001; Fig. 4b and Supplementary Fig. 15). These data indicate that the increase in Ly6C^hi^ monocytes can be attributed to their expansion or influx into the spleen. Furthermore, neutrophils from β-glucan-polymersome-treated mice expressed higher levels of activation markers MHCII and PD-L1, compared with the PBS control (*P* = 0.043 and *P* = 0.087, respectively; Supplementary Fig. 16).

To further unravel the mechanism behind the observed reduction in tumour growth, we studied the efficacy of β-glucan-polymersomes efficacy in B16F10 melanoma-bearing lymphocyte-deficient *Rag1*^−/−^ mice (intravenous injections of β-glucan-polymersomes every other day at 2.5 mg β-glucan per kg; Fig. 4c). β-Glucan-polymersomes significantly inhibited tumour growth in *Rag1*^−/−^ mice compared with the PBS control group (*P* = 0.0249; Fig. 4c), which suggest that the antitumour response directly involves innate immune cells. Furthermore, the antitumour effect of splenic myeloid cells was assessed by adoptive transfer experiments (Fig. 4d and Supplementary Fig. 15). Splenic myeloid cells from mice pretreated with β-glucan-polymersomes (intravenous injections of β-glucan-polymersomes every other day at 2.5 mg β-glucan per kg) tended to reduce tumour growth in naive mice when inoculated together with B16F10 melanoma cells, compared with the PBS control (*P* = 0.0691; Fig. 4d). However, intravenous administration of pretreated myeloid cells to B16F10 melanoma-bearing mice did not affect tumour growth, probably owing to the poor tissue-homing capacity of these cells (Supplementary Fig. 15). Interestingly, myeloid cells (CD11b^+^) isolated from the spleen of mice treated with β-glucan-polymersomes suppressed the ex vivo expansion of CD8^+^ T cells less strongly than myeloid cells from mice that received PBS injections (*P* = 0.0322; Supplementary Fig. 16). This suggests that myeloid cells may indirectly potentiate an antitumour T cell response, which is in line with the tumour growth reduction resulting from the combination with anti-PD-1 therapy (Fig. 4a). These results demonstrate substantial therapeutic efficacy of intravenously administered β-glucan-polymersomes to the B16F10 melanoma mouse model and indicate that the antitumour response is mainly driven by myeloid cells in the spleen.

## Polymersome splenic avidity is preserved across species

**Fig. 5:**
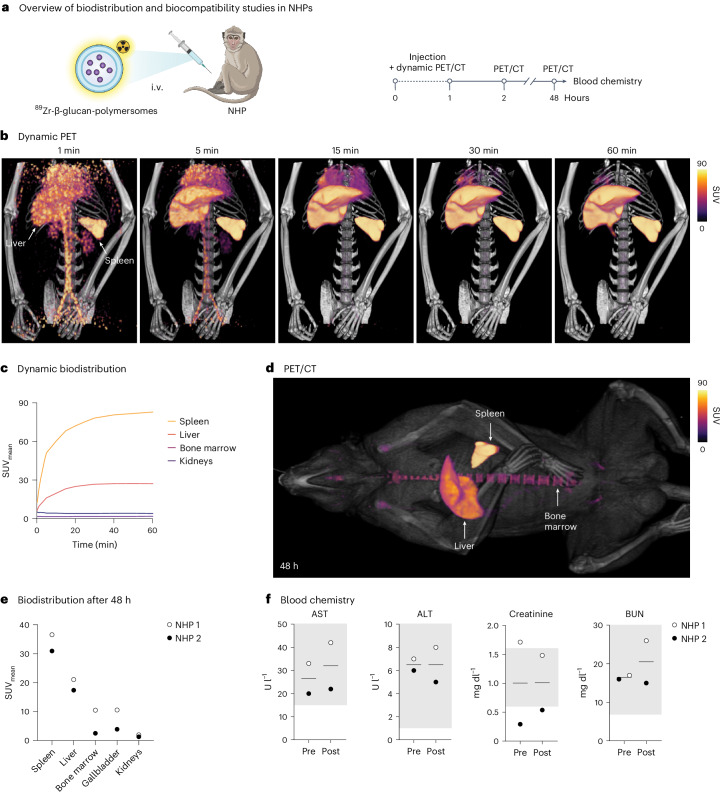
β-Glucan-polymersome biodistribution and biocompatibility studies in NHPs confirm nanocarriers’ splenic avidity across different species. **a**, Schematic representation of the study. ^89^Zr-labelled β-glucan-polymersomes were intravenously injected in two NHPs that then underwent dynamic and static PET/CT imaging. **b**, Dynamic PET/CT images at 1 min, 5 min, 15 min, 30 min and 60 min after injections, showing rapid accumulation of ^89^Zr-β-glucan-polymersomes in the spleen and liver. **c**, Quantified uptake of ^89^Zr-β-glucan-polymersomes in representative organs from images in **b**, indicating higher accumulation in the spleen than other organs, *n* = 1. **d**, PET/CT images at 48 h after injections reveal accumulation in the spleen, liver and bone marrow. **e**, Quantified uptake of ^89^Zr-β-glucan-polymersomes in representative organs at 48 h from images in **d**, *n* = 2. **f**, Blood chemistry performed on NHP serum taken before (Pre) and 48 h after (Post) ^89^Zr-β-glucan-polymersome administration, *n* = 2. The grey boxes indicate reference values^1^. Alanine transaminase (ALT), aspartate transaminase (AST), creatine and blood urea nitrogen (BUN) levels show no signs of severe toxicity. Schematic illustrations of syringe and NHP were created with BioRender.com.

We investigated the translational potential of β-glucan-polymersomes by studying their biodistribution and biocompatibility in two cynomolgus monkeys (6.2 kg and 10.2 kg). These NHPs were injected intravenouslywith ^89^Zr-β-glucan-polymersomes and imaged with whole-body PET/CT (Fig. 5a and Supplementary Video 1). Additionally, blood was collected before and after treatment to assess toxicity. Dynamic images of the first hour after injection showed rapid polymersome clearance from the blood and high accumulation in the spleen and liver (Fig. 5b,c). At 48 h after injection, we still observed notable splenic uptake as well as high accumulation in the vertebral bone marrow (Fig. 5d). β-Glucan-polymersome uptake in other organs, including the kidneys, lungs, brain and heart, was negligible at all time points (Fig. 5e and Supplementary Fig. 17). Blood chemistry revealed only minor changes in alanine transaminase, aspartate transaminase, creatinine and blood urea nitrogen, indicating that β-glucan-polymersome treatment is well tolerated^49^ (Fig. 5f). Overall, β-glucan-polymersomes’ in vivo behaviour in NHPs was similar to that in mice, showing that the favourable biodistribution of this platform is preserved across species.

## Conclusion

In the past decade, researchers have demonstrated that the spleen serves as a reservoir of immunosuppressive myeloid cells that are recruited to the tumour microenvironment and promote tumour growth^13–16^. Therefore, therapeutic targeting of splenic myeloid cells for delivery of immunomodulatory drugs holds unexplored potential for cancer treatment. Designer nanomedicines with tunable properties are of particular interest for immunotherapy purposes, especially when targeting innate immune cells is desired^28,35,38^. We present a comprehensive workflow for multiparametric assessment of the in vivo behaviour of a polymeric nanomedicine platform, facilitating the development of an immunotherapy targeting innate immunity. To efficiently target innate immune cells in the spleen, we established a small library of four polymersome nanocarriers with comparable composition but diverse topologies. Extensive studies in tumour-bearing mice revealed that polymersome biodistribution and cellular specificity are dictated by their size and administration route, but not shape. For all topologies, polymersomes swiftly accumulated in the spleen, with circulation half-lives of less than 50 min. Importantly, using a combination of flow and mass cytometry, we found large, spherical polymersomes to most prominently associate with red pulp myeloid cells in the spleen. On the basis of these results, we loaded the β-glucan laminarin in these large, spherical polymersomes and demonstrated their propensity to efficiently target myeloid cells in the spleen and bone marrow. In a proof-of-concept study, we demonstrated potent antitumour effects of β-glucan-polymersomes in a B16F10 melanoma mouse model. Using ^89^Zr-radiolabelling and in vivo PET/CT imaging on a clinical scanner, we found the β-glucan-polymersomes to show a similar splenic propensity in NHPs.

## Methods

### Polymersome formulation

#### Preparing BODIPY-, DFO- or N_3_-labelled polymersomes

PEG_22_-PDLLA_45_ and 5 wt% BODIPY-PEG_24_-PDLLA_45_, DFO-PEG_24_-PDLLA_45_ or N_3_-PEG_24_-PDLLA_45_ were weighed in a glass vial (15 ml) and a dioxane/THF mixture (4:1 v/v) was added to obtain a total concentration of 10 mg ml^−1^. The resulting solution was stirred for approximately 30 min and then transferred to a flow cabinet. The polymer solution was filtered (0.2 µm PTFE filter) into 15 ml glass vials (2 ml per vial) containing stirring bars. Subsequently, each vial was capped with a rubber septum and solutions were stirred for approximately 5 min. Using a syringe pump, Endotoxin-Free Ultra Pure Water (2 ml, 50 vol%, Chemicon, Merck) was added at a rate of 1 ml h^−1^ to form large polymersomes. Small polymersomes were formed by adding 1 ml (33 vol%) of Endotoxin-Free Ultra Pure Water to the polymer solution in dioxane/THF (4:1 v/v) to increase membrane flexibility for extrusion (downsizing process). The polymersome solution was extruded 11 times through an Avanti Mini-Extruder, containing a 100 nm polycarbonate membrane filter (Whatman Nuclepore track-etched membranes, Merck) supported by two 10 mm filter supports (Avanti Polar Lipids). All extrusion materials were extensively washed with Endotoxin-Free Ultra Pure Water before use. For both large (no extrusion) and small (extruded) polymersomes, the obtained cloudy solutions were subsequently transferred to a pre-hydrated dialysis membrane (molecular weight cut-off (MWCO) of 12,000–14,000 Da, Spectra/Por) in a flow cabinet and dialysed against precooled Milli-Q water (1 l, Merck Millipore Q-Pod system (18.2 MΩ) with a 0.22 µm Millipore Express 40 filter) at 4 °C for 24 h, with a water change after the first hour, to form LgS or SmS. To form LgT and SmT, the polymersome solutions were dialysed against a 50 mM NaCl solution instead of Milli-Q water. Finally, the spherical and tubular polymersomes were dialysed against PBS (1 l, Gibco PBS Tablets, Thermo Fisher Scientific, dissolved in Milli-Q water) at 4 °C for 24 h with a PBS change after 1 h, 4 h, 8 h and 20 h, using Endotoxin-Free Dulbecco’s PBS (Chemicon, Merck) at 8 h and 20 h. The resulting polymersome solutions were concentrated to 10 mg ml^−1^ using centrifugal filtration for approximately 10 min at 4 °C at a speed of 4,000 × *g* (10 kDa Amicon Ultra 15 ml, Merck). The concentrated polymersomes were resuspended and transferred to endotoxin-free Eppendorf vials in a flow cabinet, where they were stored at 4 °C until use.

#### ^89^Zr-radiolabelling of DFO-polymersomes

A solution of ^89^Zr oxalate in 1 M oxalic acid (3D Imaging) was diluted with PBS (100 μl) and neutralized using a 1 M sodium carbonate solution until a pH between 6.8 and 7.4 was obtained. The ^89^Zr solution (typically <150 μl) was added to the DFO-containing polymersomes (0.5–1.0 ml) and incubated at 37 °C using a thermomixer (600 rpm) for 45 min. The resulting solution was purified using a PD-10 desalting column (GE) with PBS as the eluent. The radiochemical purity of the radiolabelled polymersomes was typically >95%, as assessed by radio-TLC, using iTLC-SG paper (Agilent) as the stationary phase and EDTA (50 mM) as the eluent.

#### ^157^Gd-isotope labelling of N_3_-polymersomes

^157^Gd was complexed to DO3A-DBCO as described in Supplementary Methods. DO3A(^157^Gd)-DBCO was added to the N_3_-containing polymersomes in PBS and incubated at 30 °C using a thermomixer (300 rpm) for 2 h followed by overnight incubation at room temperature using a tube rotator. To remove uncoupled DO3A(^157^Gd)-DBCO, the ^157^Gd-polymersomes were transferred to pre-hydrated dialysis membranes (MWCO 12,000–14,000 Da, Spectra/Por) and dialysed against PBS (2 l) at 4 °C for 24 h with a PBS change after the first hour.

#### Preparing β-glucan-polymersomes

To form β-glucan-loaded polymersomes, the preparation procedure for large spherical polymersomes was used with the following modifications: A solution of 20 mg ml^−1^ laminarin (Laminarin from *Laminaria digitata*, Sigma-Aldrich, Merck), dissolved in Endotoxin-Free Ultra Pure Water, was sonicated and vigorously vortexed for 5 min to ensure complete dissolution. In a flow cabinet, the solution was filtered through a sterile 0.2 µm filter (Pall Acrodisc Syringe Filters with Supor Membrane, Sterile, 0.2 µm, 25 mm). Using a syringe pump, the laminarin solution (2 ml) was added to the block copolymer solution (2 ml, 10 mg ml^−1^ PEG_22_-PDLLA_45_ **3** in dioxane/THF, 4:1 v/v) at a rate of 1 ml h^−1^, thereby inducing self-assembly of β-glucan-loaded polymersomes. The polymersomes were dialysed at 4 °C against Milli-Q water as previously described. To remove unencapsulated laminarin, the dialysed polymersomes were subsequently transferred to dialysis membranes with a larger cut-off value (MWCO 1,000,000 Da, Spectra/Por) and dialysed against Milli-Q (2 l) at 4 °C for 24 h with a water change after the first hour. Finally, polymersomes were dialysed against PBS (2 l) at 4 °C for 24 h with a PBS change after 1 h, 4 h and 8 h, with the final PBS being endotoxin-free. The resulting purified β-glucan-polymersome solutions were concentrated to 40 mg ml^−1^ using centrifugal filtration at 15 °C and a speed of 2,000 × *g* (MWCO 100,000 Da, Sartorius Vivaspin Turbo 15 PES Centrifugal Concentrators). The concentrated polymersomes were resuspended and transferred to endotoxin-free tubes at 4 °C to be stored until use. β-Glucan encapsulation quantification is described in Supplementary Methods.

#### Preparing unloaded polymersomes

Unloaded polymersomes were formulated as controls using the same procedure as for preparing β-glucan-polymersomes, using Endotoxin-Free Ultra Pure Water without laminarin for polymersome self-assembly.

#### Preparing DFO-labelled β-glucan-polymersomes

DFO-labelled β-glucan-polymersomes were formulated as described for β-glucan-polymersomes but replacing 5 wt% of PEG_22_-PDLLA_45_ with DFO-PEG_24_-PDLLA_45_.

### Animals

Female 8-week-old *Rag1*^−^^/^^−^ (B6.129S7-*Rag1*^*tm1Mom*^/J) and C57BL/6 mice were purchased from The Jackson Laboratory. For NHP experiments, two adult male 14- and 15-year-old cynomolgus monkeys (*Macaca fascicularis*) were used. All animals had free access to food and water. Mice were co-housed in climate-controlled rooms (ambient temperature and humidity) with 12 h light/dark cycles. The mice were allowed to acclimate to the housing facility for at least 1 week before they were randomly assigned to experimental groups. NHPs were pair-housed, when possible, in climate-controlled conditions with 12 h light/dark cycles. All animal experiments were performed in accordance with Icahn School of Medicine at Mount Sinai Institutional Animal Care and Use Committee (IACUC) and VU University Medical Center Dierexperimentencommissie (DEC) guidelines as well as Dutch requirements and laws on animal experimentation.

### B16F10 melanoma model

The B16F10 cancer cell line (B16-F10, ATCC, CRL-6475) was kindly provided by I. J. Fidler (MD Anderson Cancer Center) and grown in Dulbecco’s modified Eagle’s medium supplemented with 10% heat-inactivated fetal bovine serum (FBS), 100 IU ml^−1^ penicillin and 100 μg ml^−1^ streptomycin. Eight-week-old female C57BL/6 mice were subcutaneously injected with 10^5^ B16F10 cells in PBS (100 μl), supplemented with 0.5% FBS. Biodistribution and cellular specificity studies were performed 11 days after tumour inoculation. For therapeutic studies, tumour growth was assessed daily by caliper measurement. The tumour volume was calculated as (width × width × height) × 0.52. The maximal permitted tumour size was 2,000 mm^2^, which was not exceeded. Mice were euthanized when humane endpoints were reached.

### ^89^Zr-polymersome PET/CT imaging in mice

^89^Zr-polymersomes were administered to female C57BL/6 mice bearing B16F10 tumours. Mice were either intravenously injected with 2.14 ± 0.54 MBq ^89^Zr-polymersomes in PBS (200 μl) or injected subcutaneously in the footpad, on the ipsilateral side of the tumour, with 0.51 ± 0.12 MBq ^89^Zr-polymersomes in PBS (30 μl). Forty-eight hours after injection, mice were anaesthetized with isoflurane (Baxter Healthcare)/oxygen gas mixture (2% for induction, 1% for maintenance) and whole-body PET and CT scans were performed using a nanoScan PET/CT system (Mediso Imaging Systems). CT imaging was conducted during infusion of an iodine-based contrast agent (Iopamidol, ISOVUE-M, Bracco Diagnostics) to better visualize the vasculature and organs. CT settings were voltage and current of 50 kVp and 600 μA, exposure time of 300 ms per frame and 480 projections. PET scans were 25 min long. The energy window applied was 400–600 keV and the image data were normalized to correct for non-uniform PET response. Scans were reconstructed into three-dimensional volumes with a voxel dimension of 0.4 × 0.4 × 0.4 mm^3^, with the Tera-Tomo 3D iterative reconstruction algorithm, and the iteration and subset numbers were 4 and 6, respectively.

### ^89^Zr-polymersome PET/CT imaging in NHPs

After an overnight fast, monkeys were anaesthetized with ketamine (5.0 mg kg^−1^) and dexmedetomidine (0.0075–0.015 mg kg^−1^), and blood was collected from the femoral vein. The animals were injected with ^89^Zr-polymersomes (1 mCi and 0.5 mCi dose, 1.7 and 0.9 ml 0.59 mCi ml^−1^, laminarin dose 0.07 and 0.06 mg kg^−1^, for 10 and 6 kg animals, respectively). Dynamic PET imaging was performed during the first 60 min after infusion. Additional PET/CT scans were performed at 1 h and 48 h after injection. PET and CT images were acquired on a combined PET/CT system (Biograph Vision, Siemens Healthineers). CT imaging parameters were as follows: X-ray tube current of 61 mAs, exposure of 38 mAs, exposure time of 500 msec and a spiral pitch factor of 0.8. After dynamic PET image acquisition, static whole-body PET images were acquired from the cranium to the pelvis using 4 consecutive bed positions of 15 min each. Before PET acquisition, whole-body CT was acquired. After acquisition, PET raw data from each bed were reconstructed and collated together offline using the Siemens proprietary e7tools with an ordered subset expectation maximization algorithm with point spread function correction. A dual-compartment (soft tissue and air) attenuation map was used for attenuation.

### Imaging-based analyses of ^89^Zr-polymersome distribution in NHPs

Image analyses were performed using Osirix MD version 12.0. Whole-body CT images were fused with PET images and analysed in a coronal plane. Regions of interest (ROIs) were drawn on various tissues. The spleen, liver, bone marrow, gallbladder and kidneys were traced in their entirety, with bone marrow sampled from the femur and three vertebrae. Mean standardized uptake values (SUVs) were calculated for each ROI. Subsequently, polymersome uptake in each organ was expressed as the average of all mean SUVs per organ.

### Gamma counting

To assess ^89^Zr-polymersome pharmacokinetics, blood samples were collected at 1 min, 5 min, 15 min and 30 min and 1 h, 2 h, 6 h, 24 h and 48 h after intravenous administration. To determine ^89^Zr-polymersome biodistribution, mice were euthanized directly after PET imaging and tissues were collected for ex vivo gamma counting. Blood and tissue samples were weighed and counted on a Wizard^2^ 2470 Automatic Gamma Counter (PerkinElmer). The radioactivity content was decay corrected and expressed as the percentage injected dose per gram of tissue (%ID g^−1^). Blood radioactivity content was fitted with a two-phase decay function. The weighted blood half-life was calculated as (*t*_1/2_ fast × % fast + *t*_1/2_ slow × % slow)/100.

### β-Glucan-polymersome treatment in mice

Mice were treated intravenously through lateral tail vein injection with β-glucan-polymersomes at either 5 mg kg^−1^ or 2.5 mg kg^−1^ on day 7 and at 2.5 mg kg^−1^ on days 9, 11, 13 and 15 after tumour inoculation.

### Checkpoint inhibitor therapy

On days 9, 12 and 15 after tumour inoculation, mice were intraperitoneally injected with anti-PD-1 (200 μg, clone RMP1-14, BioXcell) in PBS (200 μl).

### Adoptive transfer experiments

For intratumoural adoptive transfer, naive mice were treated with either PBS or 2.5 mg kg^−1^ for 5 injections in 8 days. The day after the last treatment, spleens were collected from these mice and CD11b^+^ myeloid cells were sorted using CD11b magnetic beads according to the manufacturer’s protocol. For one experiment, naive mice were shaved and then subcutaneously inoculated with a mixture of 50,000 B16F10 melanoma cells and 50,000 CD11b^+^ myeloid cells from mice either treated with PBS or polymersomes. For another experiment, mice were inoculated with 100,000 B16F10 melanoma cells until palpable tumours formed. Then mice were randomized and intravenously injected with 500,000 CD11b-positive myeloid cells from the PBS group or the polymersome group. For intravenous adoptive transfer, mice were inoculated with B16F10 melanoma cells and 500,000 splenic myeloid cells from pretreated mice were injected intravenously through lateral tail vein injection.

### Flow cytometry

To study cellular specificity, mice were injected with BODIPY-polymersomes 48 h before flow cytometry analysis (intravenously 100 μl, or subcutaneously 25 μl, of 10 mg ml^−1^ BODIPY-polymersomes). Animals were euthanized and perfused with cold PBS (20 ml). Femurs, lymph nodes and spleens were collected and stored on ice. Bone marrow cells were flushed out of femurs and strained through a 70 μm strainer. Lymph nodes and spleens were fragmented and meshed through a 70 μm strainer. Bone marrow and spleen samples were incubated with lysis buffer and washed with FACS buffer (Dulbecco’s PBS complemented with 1% FBS, 1 mM EDTA, 0.5% bovine serum albumin and 0.1% NaN_3_). For β-glucan-polymersome therapeutic studies, spleen single-cell suspensions were incubated with anti-CD115 (clone AFS98), anti-Ly6C (clone Al-21), anti-Ly6G (clone 1A8), anti-CD11b (clone M1/70), anti-CD19 (clone 1D3) and anti-CD90.2 (clone 53-2.1). Single-cell suspensions were incubated with anti-CD45 (clone 30-F11), anti-Ly6C (clone AL-21), anti-CD11b (clone M1/70), anti-CD11c (clone N418), anti-F4/80 (clone BM8), and a Lineage cocktail containing anti-CD90.2 (clone 53.2-1), anti-Ter119 (clone TER-119), anti-NK1.1 (clone PK136), anti-CD49b (clone DX5), anti-CD45R (clone RA3-6B2) and anti-Ly6G (clone 1A8). To study ex vivo marker expression and in vitro T cell suppression, single-cell suspensions were incubated with anti-CD45 (clone 30-F11), anti-CD11b (clone M1/70), anti-Ly6G (clone 1A8), anti-CD115 (clone AFS98), anti-I-A/I-E (MHCII; clone M5/114.15.2), anti-PD-L1 (clone 10F.9G2), anti-CD3 (clone 17A2), anti-CD4 (clone RM4-4) and anti-CD8a (clone 53-6.7). Data were acquired on a LSRFortessa (BD Biosciences) or a CytoFLEX LX (Beckmann Coulter), and the BODIPY signal was detected in the FITC channel. Data were analysed using FlowJo v10.9.0 (Tree Star).

### Mass cytometry

To study cellular specificity, mice were injected with ^157^Gd-polymersomes 48 h before mass cytometry analysis (0.7 mg polymer per mouse). Animals were euthanized and perfused with cold PBS (20 ml). Spleens were collected and stored on ice, fragmented and meshed through a 70 μm strainer. Spleen samples were incubated with lysis buffer and washed with PBS. Spleen single-cell suspensions were incubated with anti-CD45 (clone 30-F11), anti-CD3 (clone 145-2C11), anti-CD11c (clone N418), anti-F4/80 (clone BM8), anti-Ly6C (clone HK1.4), anti-CD19 (clone 6D5), anti-Ly6G (clone 1A8), anti-Ter119 (clone TER-119), anti-CD200R3 (clone Ba160), anti-CD49b (clone DX5), anti-CD169 (clone 3D6.112), anti-CD115 (clone AFS98), anti-MARCO (clone EPR24317-33), anti-CD117 (clone 2B8), anti-NK1.1 (clone PK136), anti-CD172a (clone P84), anti-Sca-1 (clone D7) and anti-CD11b (clone M1/70) in the presence of Fc-blocker. Samples were fixed in paraformaldehyde, incubated with iridium (Ir) DNA intercalator to discriminate between live and dead cells, and spiked with internal metal isotope normalization beads before data acquisition on a Helios mass cytometry (Standard BioTools). Acquired data were normalized using the Helios Software (Standard BioTools, CyTOF Software version 7.0) and uploaded into the Cytobank web server (Cytobank) for further quality control data processing. Gaussian parameters of the Helios system were used for quality control, and doublet, dead cell and normalization bead exclusion. Data were transformed using an arcsinh(X/5) transformation. Single live intact cells were then further analysed using FlowJo v10.9.0 (Tree Star).

### Biochemistry of NHP serum

NHP blood samples were collected before and 48 h after ^89^Zr-β-glucan-polymersome administration. Blood chemistry analysis was performed on serum by IDEXX BioAnalytics.

### Statistical analysis

Data are presented as mean ± s.e.m. or as mean ± s.d., as specified in the figure captions. Differences were evaluated using a two-way analysis of variance (ANOVA) to compare immune cell specificity, a repeated measures two-way ANOVA for tumour growth, or an unpaired Mann–Whitney test (two-tailed) to compare splenic immune cells. For all tests, *P* < 0.05 represents statistical significance. Statistical analyses were performed with GraphPad Prism 10.

### Reporting summary

Further information on research design is available in the Nature Portfolio Reporting Summary linked to this article.

## Online content

Any methods, additional references, Nature Portfolio reporting summaries, source data, extended data, supplementary information, acknowledgements, peer review information; details of author contributions and competing interests; and statements of data and code availability are available at 10.1038/s41565-024-01727-w.

## Supplementary information

Supplementary Information Supplementary Methods, Tables 1–3, Figs. 1–17 and references.

Reporting Summary

Supplementary Video 1 Three-dimensional whole-body PET/CT image of a non-human primate 48 h after intravenous ^89^Zr-β-glucan-polymersome administration.

## Source data

Source Data Fig. 3 Statistical source data.

Source Data Fig. 4 Statistical source data.

## Data Availability

Raw data of the cryo-TEM analysis are publicly available on the 4TU.ResearchData repository and can be accessed through 10.4121/4f8c02c7-9787-435c-a9b2-1adc3af5dd22. Other raw data are available upon request. Other data are presented in the main text and Supplementary Information.
