# Supplementary material for: Polymersomes with splenic avidity target red pulp myeloid cells for cancer immunotherapy
Source: Nat Nanotechnol. 2024 Jul 31;19(11):1735–44. doi: 10.1038/s41565-024-01727-w (PMC11567884; doi:10.1038/s41565-024-01727-w)
Supplement: Supplementary file 1 — Supplementary Methods, Tables 1–3, Figs. 1–17 and references. [file 41565_2024_1727_MOESM1_ESM.pdf]

nature nanotechnology

Supplementary information https://doi.org/10.1038/s41565-024-01727-w

Polymersomes with splenic avidity target red pulp myeloid cells for cancer immunotherapy

In the format provided by the authors and unedited

Contents

Supplementary Methods
Supplementary Tables 1-3
Supplementary Figures 1-17
References

Supplementary Methods

Instruments

Nuclear Magnetic Resonance (NMR). Proton nuclear magnetic resonance measurements were performed on a Bruker 400 Ultrashield™ spectrometer equipped with a Bruker SampleCase autosampler and Bruker Topspin v3.6.1 software, using $CDCl_3$ as a solvent and TMS as an internal standard. Block copolymers were dissolved at a concentration of 10 mg/mL. NMR data was analyzed using MestReNova v11.0.

Gel permeation chromatography (GPC). GPC was conducted using a Shimadzu Prominence-i GPC system with a PL gel 5 µm mixed D and mixed C column (Polymer Laboratories) as well as a Shimadzu RID-20A differential refractive index detector and THF as an eluent, with a flow rate of 1 mL/min. Block copolymers were dissolved in THF at a concentration of 1 mg/mL. GPC data was analyzed using LabSolutions v5.73.

Infrared spectroscopy (IR). IR spectra of lyophilized copolymers and polymer conjugates were recorded on a PerkinElmer Spectrum One spectrometer and analyzed with PerkinElmer SpectrumIR v10.6.1.942.

Matrix-assisted laser desorption/ionization time-of-flight mass spectrometry (MALDI-TOF-MS). MALDI-TOF-MS measurements were taken with an Autoflex Speed (Bruker, Bremen Germany) instrument equipped with a smartbeam laser and capable of executing both linear and reflector modes, and Bruker flexControl v3.4 software. Two matrices were used, A-Cyano-4-hydrocycinnamic acid (CHCA) from Fluka (Zwijndrecht, The Netherlands) and 2-[(2E)-3-(4-tert-butylphenyl)-2-methylprop-2-enylidene] malonitril (DCTB). Matrix solutions were freshly prepared in Tetrahydrofuran (THF) stabilized with butylated hydroxytoluene from Biosolve. Concentration was approximately 20 mg/mL. A sample solution of 1 mg/mL in THF and a matrix solution were mixed in an Eppendorf tube, and 1 µl was pipetted onto a stainless steel MALDI target plate and allowed to dry. Mass spectra were acquired in the linear positive ion mode by summing spectra from 500 random laser shots at an acquisition rate of 500 Hz.

Differential scanning calorimetry (DSC). To determine the glass transition temperature (T_g) of the block copolymers, DSC data were collected on a DSC Q2000, from TA instruments (Universal Analysis 2000 v4.5A software), calibrated with an indium standard. The lyophilized copolymers (4–8 mg) were portioned by weight directly into aluminum pans which were then hermetically sealed. The samples were initially heated to 20°C and then subjected to a heating and cooling cycle spanning from 80°C to -20°C, at a scanning rate of 5°C/min. This was followed by three cooling/heating cycles, from -20°C to 80°C, at scanning rates 5, 10, and 40 °C/min, respectively. The T_g was determined at the inflection point from the last heating/cooling cycle.

Radio-Thin layer chromatography (Radio-TLC). Radio-TLC of ^{89}Zr-labeled polymersomes was performed on a LabLogic Scan-Ram system. TLCs were developed by applying approximately 20 µCi of activity to iTLC-SG glass microfiber paper (Agilent Technologies) and eluting this using a 50 mM aqueous EDTA solution. Under these conditions, bare ^{89}Zr has a retention factor of close to 1 and the radiolabeled polymersomes remain at the baseline.

Dynamic Light Scattering (DLS) and Zeta (ζ) potential measurements. DLS and ζ potential measurements were performed on a Malvern Zetasizer instrument (model Nano ZSP). ZetaSizer Software v7.13 was used to process and analyze the data. For all DLS measurements, 20 µL of polymersome sample (2.5 mg/mL polymer) was diluted 10 times by mixing with 180 µL PBS (final concentration 0.25 mg/mL polymer). Measurements were conducted at 25°C using a ZEN0040 type disposable cuvette cell. Three measurements were averaged to analyze the hydrodynamic diameter (D_h = Z-average diameter) and polydispersity index (PDI). For the zeta potential measurements, 100 µL of polymersome sample (2.5 mg/mL polymer) in PBS was diluted 10 times by mixing with 900 µL Milli-Q water (final concentration 0.25 mg/mL polymer). Measurements were conducted at 25°C using a DTS1070 folded capillary cell at 150V. Three measurements, at intervals of 2 min, were averaged to calculate the mean ζ potential. Zeta deviation was reported as the standard deviation.

Nanoparticle Tracking Analysis (NTA). To track particle numbers and measure their hydrodynamic size distributions, a Nanosight NS300 instrument (Malvern Panalytical) equipped with a scientifically complementary metal–oxide–semiconductor (sCMOS) camera was used. The camera was mounted on an optical microscope to visualize the light scattered by the injected particles that were present in the focus of an 80 µm beam generated by a single-mode laser diode with a blue laser (488 nm). Small and large polymersomes (2.5 mg/mL) were diluted 2000 and 500 times in PBS buffer, respectively, so that the number of particles in the field of view was in the recommended range of 20-100, and a 1 mL volume was injected into the Nanosight chamber. For all measurements, 5 captures of 60 seconds were recorded, with a screen gain of 9 and a camera level of 11; during the data analysis using Nanosight NTA 3.2 the screen gain was set to 9 with a detection threshold of 5.

Cryogenic transmission electron microscopy (cryo-TEM). Experiments were performed using the TU/e CryoTitan (Thermo Fisher Scientific) equipped with a field emission gun operating at 300 kV, an autoloader station, and a post-column Gatan energy filter (Gatan GIF 2002, 20 eV energy slit). Samples for cryo-TEM were prepared by glow-discharging the grids (Lacey carbon-coated grids, R2/2, Cu, 200 mesh, EM sciences and Quantifoil Jena grids, R2/2, Cu, Quantifoil Micro Tools GmbH) in a Cressington 208 carbon coater for 40 seconds. Next, 3 µL of the polymersome solution (2.5 mg/mL) was pipetted onto the grid and blotted in a Vitrobot MARK IV ((Thermo Fisher Scientific)) at room temperature and 100% humidity. The grid was blotted for 3 seconds (offset: -3) and then directly plunged into and frozen in liquid ethane. Cryo-TEM images were acquired via a post-GIF 2k Gatan CCD (charge-coupled device) camera and Digital Micrograph v1.85.1535 software. TEM images were processed using ImageJ v2.0.0, a program developed by NIH and publicly available at http://rsbweb.nih.gov/ij/. Polymersome dimensions were measured manually using ImageJ. Aspect ratios were calculated by comparing the measured length and width (L/W) of each polymersome.

Asymmetric Flow Field-Flow Fractionation – UV/VIS – multi-angle light scattering – quasi elastic light scattering (AF4-UV-MALS-QELS). AF4-UV-MALS-QELS measurements were performed on a Wyatt Dualtec AF4 instrument connected to a Shimadzu LC-2030 Prominence-i system with a Shimadzu LC-2030 autosampler. All fractionations were conducted on an AF4 short channel equipped with regenerated cellulose (RC) 10 kDa membrane (Millipore) and 350 µm spacer. The applied flow conditions are in **Supplementary Table 3**. The AF4 was connected to a Wyatt Optilab rEX refractive index detector and a Wyatt DAWN HELEOS II light scattering detector (MALS) installed at different angles (12.9°, 20.6°, 29.6°, 37.4°, 44.8°, 53.0°, 61.1°, 70.1°, 80.1°, 90.0°, 99.9°, 109.9°, 120.1°, 130.5°, 149.1°, and 157.8°). The MALS laser operated at 658 nm. Detectors were normalized using Bovine Serum Albumin (BSA). Light scattering data and radius of gyration (R_g) calculations were processed and analyzed with Astra v7.3.2software. As particles had radii varying from approximately 50 nm to 150 nm, the Debye model with a fit degree of 2 was used for analysis, as recommended by the manufacturer for particles across a wide size range.

Polymer synthesis and characterization

*Synthesizing and characterizing poly(ethylene glycol) – poly($_{D,L}$-Lactide) (PEG_{22}-$PDLLA_{45}$ **3**) block copolymer.* PEG_{22}-$PDLLA_{45}$ **3** was synthesized according to our previously reported procedure[1] (the synthesis scheme is outlined in **Supplementary Fig. 1a**). A 50 mL round-bottom flask (RBF) equipped with a stirring bar was dried using a heat gun under vacuum, followed by three flushing cycles with argon and vacuuming. Thereafter, the RBF was subjected to a constant argon flow. Monomethoxy-PEG-OH **1** (mPEG, 0.194 g, 0.2 mmol, poly(ethylene glycol) 1K, JenKem technology, lyophilized) and $_{D,L}$-Lactide **2** (DLL, 1.3 g, 9 mmol, Acros) were added to the dried RBF. Subsequently, dry toluene (~50 mL) was added using an argon-flushed syringe and the solvent was evaporated to dry the reagents. The rotary evaporator was flushed with an argon balloon after solvent evaporation in order to maintain an inert atmosphere after drying, and this toluene evaporation cycle was repeated once more. The dried reagents were then re-dissolved in dry DCM (13 mL; [DLL monomer] = 0.7M) using an argon-flushed syringe before DBU **3** (15 µL, 0.5 equivalents relative to [mPEG macroinitiator]) was added using an argon-flushed organic solvent pipette. The reaction was stirred at 25°C for 2 hours until ^{1}H NMR analysis showed full conversion of the $_{D,L}$-Lactide monomer. Subsequently, the reaction mixture was diluted with DCM (25 mL) and washed twice with 1M $KHSO_4$, once with Milli-Q, and once with brine (100 mL each); all washing steps were performed via extraction. The organic solution was dried using Na_2SO_4. After evaporating most of the solvent, the concentrated copolymer solution was precipitated into ice-cold diethyl ether (200 mL). The resulting waxy solid was partially dried under argon, dissolved in dioxane, and lyophilized to yield a white powder (Yield = 1.2 gram, □80% conversion by mass). ^{1}H NMR ($CDCl_3$, 400 MHz, δ in ppm): 5.18 (ttd, 89H, lactide CH, *a*), 3.64 (s, 88H, PEG backbone, *c*), 3.38 (s, 3H, Me, *d*), 1.57 (m, 270H, lactide CH_3, *b*), see **Supplementary Fig. 2a** for spectrum. GPC analysis revealed a PDI of 1.1 (**Supplementary Fig. 3a**) and DSC analysis a T_g of 22°C (**Supplementary Fig. 3b**).

*Synthesizing and characterizing N_3-PEG_{24}-$PDLLA_{45}$ **5** block copolymer.* Using a procedure similar to the ring-opening polymerization described above, azido-dPEG^{TM}(24)-OH **4** (220 mg, 0.2 mmol, Iris Biotech GmbH) and $_{D,L}$-lactide **2** (1300 mg, 9 mmol) were used to synthesize azido-functionalized block copolymer N_3-PEG_{24}-$PDLLA_{45}$ **5** (synthesis scheme is outlined in **Supplementary Fig. 1b**), yielding a white powder (Yield = 1.0 g, □70% conversion by mass). ^{1}H-NMR ($CDCl_3$, 400 MHz, δ in ppm): 5.18 (m, 90H, lactide CH, *a*), 3.72 – 3.57 (m, 94H, PEG backbone, *c*), 3.39 (t, 2H, CH_2-N_3, *r*), 1.61 – 1.49 (m, 270H, m, lactide CH_3, *b*), see **Supplementary Fig. 2b** for spectrum. GPC analysis revealed a PDI of 1.3 and UV absorbance at λ = 216 nm, indicating the presence of azido-functionality (**Supplementary Fig. 3a**). DSC analysis revealed a T_g of 18°C (**Supplementary Fig. 3b**). IR analysis showed absorption bands at characteristic wavenumbers for functional groups including hydroxyl, alkanes, and esters. Of particular interest is the absorption band observed at wavenumber 2107 cm^{-1}, which is characteristic of azido-functionality (**Supplementary Fig. 3c**). MALDI-TOF-MS revealed a $1H^+$ mass of 7537 g/mol ± multiplicities of 72 g/mol, corresponding to different degrees of polymerization.

*Synthesizing and characterizing BODIPY-PEG_{24}-$PDLLA_{45}$ **7** block copolymer-conjugate.* Using a strain-promoted azide-alkyne cycloaddition reaction (SPAAC), BODIPY-DBCO **6** (BDP FL DBCO, Lumiprobe GmbH) was covalently coupled to the N_3-PEG_{24}-$PDLLA_{45}$ **5** polymer (synthesis scheme is outlined in **Supplementary Fig. 1c**). BODIPY-DBCO **6** (23 mg, 39 µmol, 1.5 equivalents) and N_3-PEG_{24}-$PDLLA_{45}$ **5** (200 mg, 26 µmol, 1 equivalent) were weighed in an RBF (10 mL) and dissolved in DCM (3 mL, concentration 74 mg/mL). The reaction was stirred for 24 hours under argon at 25°C. The solution was diluted with DCM (10 mL) and unconjugated BODIPY-DBCO **6** was removed by acid-base work-up until the water layer was clear: once with 1M HCl and 1M NaOH, twice with 1M $KHSO_4$, once with Milli-Q, and once with brine (50 mL each). The solution was dried using Na_2SO_4. After evaporating most of the solvent, the concentrated BODIPY-copolymer solution was precipitated into ice-cold diethyl ether (200 mL). The resulting waxy solid was partially dried under argon, dissolved in dioxane, and lyophilized to yield BODIPY-PEG_{24}-$PDLLA_{45}$ **7** as an orange powder (Yield = 148 mg, □70% conversion by mass). ^{1}H NMR ($CDCl_3$, 400 MHz, δ in ppm): 7.85 – 7.35 (m, 8H, *d*), 7.12 (d, 1H, *n*), 6.87 (d, 1H, *m*), 6.28 (t, 1H, *i*), 6.12 (s, 1H, *p*), 6.05 (d, 1H, *l*), 5.18 (dddd, 87H, *a*), 3.87 (m, 4H, *j,f*), 3.64 (d, 94H, *c*), 3.27 (q, 2H, *r*), 2.66 – 2.58 (m, 2H, *k*), 2.55 (s, 3H, *q*), 2.26 (s, 3H, *o*), 1.74 (d, 4H), 1.57 (p, 304H, *b*), 0.93 (dd, 6H, *g*), see **Supplementary Fig. 2c** for spectrum. GPC analysis revealed a PDI of 1.3 and UV absorbance at λ = 254 nm and λ = 508 nm, which demonstrated the presence of DBCO and BODIPY aromatic groups and the characteristic absorbance of the fluorescent BODIPY-dye, respectively (**Supplementary Fig. 3a**). IR analysis of the resulting copolymer conjugates showed disappearance of the azido absorbance band at 2107 cm^{-1}. In addition, characteristic absorbance bands appeared at 1700-1500 cm^{-1}, indicating tetrasubstituted/cyclic alkene and thereby confirming the successful conjugation (**Supplementary Fig. 3c**). MALDI-TOF-MS revealed a $1H^+$ mass of 8129 g/mol ± multiplicities of 72 g/mol, corresponding to different degrees of polymerization. The mass difference of 592 g/mol compared to N_3-PEG_{24}-$PDLLA_{45}$ **5** matches the added mass of BODIPY-DBCO **6**.

*Synthesizing and characterizing DFO-PEG_{24}-$PDLLA_{45}$ **9** block copolymer-conjugate.* Using a SPAAC reaction, deferoxamine-DBCO **8** (DFO-DBCO, Macrocyclics™) was covalently coupled to the N_3-PEG_{24}-$PDLLA_{45}$ **5** polymer (synthesis scheme is outlined in **Supplementary Fig. 1d**). DFO-DBCO **8** (33 mg, 39 µmol, 1.5 equivalents) and N_3-PEG_{24}-$PDLLA_{45}$ **5** (200 mg, 26 µmol, 1 equivalent) were weighed in an RBF (10 mL) and dissolved in DMSO (2 mL, concentration 117 mg/mL). The reaction was stirred for 24 hours under argon in an oil bath at 50°C. The solution was lyophilized and subsequently re-dissolved in DCM (10 mL) and filtered to remove unconjugated DFO-DBCO **8**. After evaporating most of the solvent, the concentrated DFO-copolymer solution was precipitated into ice-cold diethyl ether (200 mL). The resulting waxy solid was partially dried under argon, dissolved in dioxane, and lyophilized to yield DFO-PEG_{24}-$PDLLA_{45}$ **9** as a white powder (Yield = 206 mg, □90% conversion by mass). ^{1}H NMR ($CDCl_3$, 400 MHz, δ in ppm): 10.16 – 9.43 (m, 2H, *l*), 7.97 – 7.28 (m, 8H, *d*), 7.22 – 6.64 (m, 2H, *h*), 5.18 (ttd, 84H, *a*), 3.77 – 3.58 (m, 94H, *c*), 3.57 (s, 6H, *i*), 3.15 (d, 6H, *k*), 2.90 – 2.68 (m, 4H, *p*), 2.58 (t, 4H, *o*), 2.17 (s, 3H, *q*), 1.81 – 1.44 (m, 316H, *b*), 1.48 – 1.05 (m, 18H, *j*), see **Supplementary Fig. 2d** for spectrum. GPC analysis revealed a PDI of 1.3 and UV absorbance at λ = 254 nm, which demonstrated the presence of DBCO aromatic groups (**Supplementary Fig. 3a**). IR analysis of the resulting copolymer-conjugates showed disappearance of the azido absorbance band at 2107 cm^{-1}. In addition, characteristic absorbance bands appeared at 1700-1500 cm^{-1}, indicating tetrasubstituted/cyclic alkene, thereby confirming the successful conjugation (**Supplementary Fig. 3c**). MALDI-TOF-MS revealed a $1H^+$ mass of 8383 g/mol ± multiplicities of 72 g/mol, corresponding to different degrees of polymerization. The mass difference of 847 g/mol compared to N_3-PEG_{24}-$PDLLA_{45}$ **6** matches the added mass of DFO-DBCO **4**.

Polymersome characterization

Stability test of tubular polymersomes. Large and small tubes' stability was tested under physiological conditions (**Supplementary Fig. 6b** and c). To do this, DMEM solution (Dulbecco's Modified Eagle Medium, Gibco) supplemented with 1% (w/v) streptomycin, 1% (w/v) penicillin, and 10% (w/v) FCS (Fetal Calf Serum) was added to the small and large tubes (polymersome solution:DMEM = 1:1 or 1:4 (v/v) so that the polymer concentration was 1.25 or 2.50 mg/mL with 5.0% or 7.5% FCS, respectively). The mixture was incubated at 37°C while being shaken at 500 rpm. The tubular polymersomes' hydrodynamic diameters and polydispersities were measured using DLS after 0, 1, 4, 24, and 48 hours of incubation (**Supplementary Fig. 6a**). The samples were diluted (5x) with PBS prior to DLS measurements to minimize the effect of serum proteins, and measurements were performed in triplicate. After approximately 18 hours of incubation, tubular morphology was studied using cryo-TEM imaging (**Supplementary Fig. 6b**).

UV/Vis analysis of BODIPY-polymersomes. UV/Vis analysis verified equal degree of BODIPY labeling for the four BODIPY-polymersomes (10 mg/mL polymer) (**Supplementary Fig. 6a**). Absorbance at λ = 508 nm was determined using a NanoDrop 1000 spectrophotometer (Thermo Scientific). Measurements were performed in triplicate.

^{157}Gd-complexation to DO3A-DBCO. Isotopically enriched (92.3%) $^{157}GdCl_3 \cdot 6H_2O$ (Trace Sciences International) was dissolved in Milli-Q water and complexed with DO3A-DBCO (Macrocyclics Inc.) at pH 6,5 for 72 hours at 80°C. Complete complexation was verified using a previously described xylenol orange-based colorimetric assay[2].

ICP-MS sample preparation. An equal volume of 65% nitric acid was added to the ^{157}Gd-polymersomes and the samples were incubated at 80°C using a thermomixer (300rpm) for 1 hour. Subsequently, the samples were diluted in 5mL Milli-Q water and the ^{157}Gd content was determined with ICP-MS.

Quantifying of β-glucan encapsulation. A phenol-sulfuric acid colorimetric assay (Total Carbohydrate Assay Kit, Sigma-Aldrich, Merck) was used to quantify laminarin concentration encapsulated in the polymersomes. Polymersomes and polymers were completely removed to circumvent unwanted interference with the assay. Briefly, 20 µL of concentrated polymersome sample (40 mg/mL polymer) was lyophilized in a PCR Eppendorf tube with an additional punched lid. The resulting powder was subsequently dissolved in 100 µL Milli-Q water, then sonicated and vortexed to ensure laminarin dissolution. Subsequently, the suspension was centrifuged for 5 minutes to remove the insoluble polymers (Mini Microcentrifuge with a PCR Eppendorf tube-holder). The supernatant was transferred to a 1.5 mL Eppendorf tube and the centrifugation steps were repeated twice at 13,000xg for 5 minutes. Finally, 30 µL of the supernatant was used, according to the assay protocol. Standard curves were prepared using 0, 4, 8, and 12 µL of a laminarin solution in Milli-Q water (1 mg/mL), plus 6 µL PBS (to compensate for the salt present in the samples) and additional Milli-Q water to a final volume of 30. Polymersome concentration filtrates were used as a negative control and laminarin-spiked unloaded polymersomes as a positive control. Absorbance measurements were performed on a Tecan Spark 10M multimode platereader and Excel Microsoft 365 was used to analyze the data. The laminarin amount (µg) in each sample was determined by interpolating from the standard curves and then converting to the sample concentration in mg/mL (conversion factor = 5 (dilution factor)/30 (sample volume)/1000 (µL)). The encapsulation efficiency (ee%) was calculated using equation (1). All samples and standards were assayed in triplicate.

$$(1)\ encapsulation\ efficiency\ (ee\%) = \frac{\frac{[Laminarin]_{initial}}{[Polymer]_{initial}}}{\frac{[Laminarin]_{final}}{[Polymer]_{final}}} = \frac{\frac{5\ mg/mL\ Laminarin}{10\ mg/mL\ Polymer}}{\frac{x\ mg/mL\ Laminarin}{40\ mg/mL\ Polymer}} \cdot 100\%$$

T cell suppression assay. Animals were euthanized and perfused with cold PBS (20 mL). Spleens were collected and stored on ice. Spleens were fragmented and meshed through a 70-µm strainer and incubated with lysis buffer and washed with MACS buffer (Dulbecco's PBS complemented with 2mM EDTA and 5% bovine serum albumin). $CD11b^+$ cells and T cells were isolated with CD11b MicroBeads and the mouse Pan T Cell Isolation Kit II, respectively according to the manufacturer's protocol (Miltenyi Biotec). T cells were stained with the CellTrace™ Violet proliferation kit according to the manufacturer's protocol (Thermo Fisher Scientific Inc.) and co-cultured at different ratios with isolated $CD11b^+$ cells. Cells were incubated for three days in Roswell Park Memorial Institute (RPMI) 1640 medium supplemented with 10% heat-inactivated fetal bovine serum (FBS),

100 IU/mL penicillin, and 100 μg/mL streptomycin, and stimulated with Dynabeads (1:1 bead:cell ratio) and 50IU/mL of recombinant human IL-2. $CD8^+$ T cell proliferation was assessed by flow cytometry.

Supplementary Tables

Supplementary Table 1 │DFO- and BODIPY-polymersome characterization by dynamic light scattering (DLS), nanoparticle tracking analysis (NTA) and asymmetric flow field-flow fractionation coupled with multi-angle static and quasi-elastic light scattering (AF4-MALS-QELS). Hydrodynamic diameter (D_{h_DLS}), polydispersity index (PdI) and ζ potential were measured with DLS (n=3). Hydrodynamic diameter (D_{h_NTA}) and particle concentration were measured with NTA. Radius of gyration (R_g = Root Mean Square (RMS) radius) and hydrodynamic radius (R_h) were determined by MALS and QELS, respectively. Shape factor ρ is defined as R_g/R_h. D_{h_NTA}, ζ -potential, R_g and R_h are presented as mean ± SD, and particle concentration is presented as mean ± SE.

Name	D_{h_DLS} (nm)	PDI	ζ-potential (mV)	D_{h_NTA} (nm)	Concentration (particles/mL)	R_g (nm)	R_h (nm)	ρ (R_g/R_h)
**BODIPY-SmS**	132	0.04	-30 ±5	141 ±29	$1.8{\cdot}10^{12}$ ±$4.1{\cdot}10^{10}$	38 ±7	39 ±2	0.9±0.1
**BODIPY-SmT**	129	0.10	-30 ±6	150 ±40	$2.1{\cdot}10^{12}$ ±$3.7{\cdot}10^{10}$	50 ±10	39 ±2	1.3±0.2
**BODIPY-LgS**	329	0.08	-36 ±4	289 ±80	$2.3{\cdot}10^{11}$ ±$3.4{\cdot}10^{9}$	105 ±1	89 ±5	1.2±0.1
**BODIPY-LgT**	362	0.06	-37 ±6	312 ±75	$1.5{\cdot}10^{11}$ ±$1.0{\cdot}10^{10}$	111 ±1	77 ±5	1.4±0.1
**DFO-SmS**	108	0.05	-21 ±9	120 ±22	$2.0{\cdot}10^{12}$ ±$4.2{\cdot}10^{10}$	45 ±6	42 ±2	1.1±0.1
**DFO-SmT**	106	0.07	-23 ±5	120 ±30	$2.3{\cdot}10^{12}$ ±$7.1{\cdot}10^{10}$	50 ±11	39 ±2	1.3±0.2
**DFO-LgS**	377	0.04	-30 ±5	324 ±83	$1.6{\cdot}10^{11}$ ±$7.2{\cdot}10^{9}$	103 ±0.2	84 ±4	1.2±0.1
**DFO-LgT**	388	0.03	-30 ±5	346 ±78	$1.8{\cdot}10^{11}$ ±$1.1{\cdot}10^{10}$	112 ±2	87 ±7	1.3±0.1

Supplementary Table 2 │ β-glucan-loaded and unloaded polymersome characterization by DLS and NTA. Hydrodynamic diameter (D_{h_DLS}), polydispersity index (PDI), and ζ potential were determined with DLS. Hydrodynamic diameters (D_{h_NTA}) and particle concentrations were measured with NTA. ζ Potential and D_{h_NTA} are presented as mean ± SD, and particle concentration is presented as mean ± SE.

Name	D_{h_DLS} (nm)	PDI	ζ-potential (mV)	D_{h_NTA} (nm)	Concentration (particles/mL)
DFO-β-glucan-polymersomes	334	0.15	-21 ±5	284 ±103	$2.1{\cdot}10^{11}$ ±$1.1{\cdot}10^{10}$
β-glucan-polymersomes	332	0.15	-24 ±4	287 ±92	$1.9{\cdot}10^{11}$ ±$1.1{\cdot}10^{10}$
Unloaded polymersomes	326	0.20	-21 ±9	312 ±89	$1.9{\cdot}10^{11}$ ±$8.0{\cdot}10^{9}$

Supplementary Table 3 │ Asymmetric flow field-flow fractionation (AF4) method. The applied flow conditions were 1.50 mL/min detector flow, 1.50 mL/min focus flow, and 0.20 mL/min injection flow.

Start (min)	End (min)	Mode	Cross flow start (mL/min)	Cross flow end (mL/min)
**0**	1	Elution	0.00	3.00
**1**	3	Elution	3.00	3.00
**3**	4	Focus	-	-
**4**	9	Focus + Inject	-	-
**9**	14	Elution	3.00	3.00
**14**	16	Elution	3.00	1.17
**16**	18	Elution	1.17	0.49
**18**	20	Elution	0.49	0.24
**20**	23	Elution	0.24	0.10
**23**	38	Elution	0.10	0.00
**38**	39	Elution	0.00	0.00
**39**	40	Elution + Inject	0.00	0.00
**40**	45	Elution	0.00	0.00

Supplementary Figures

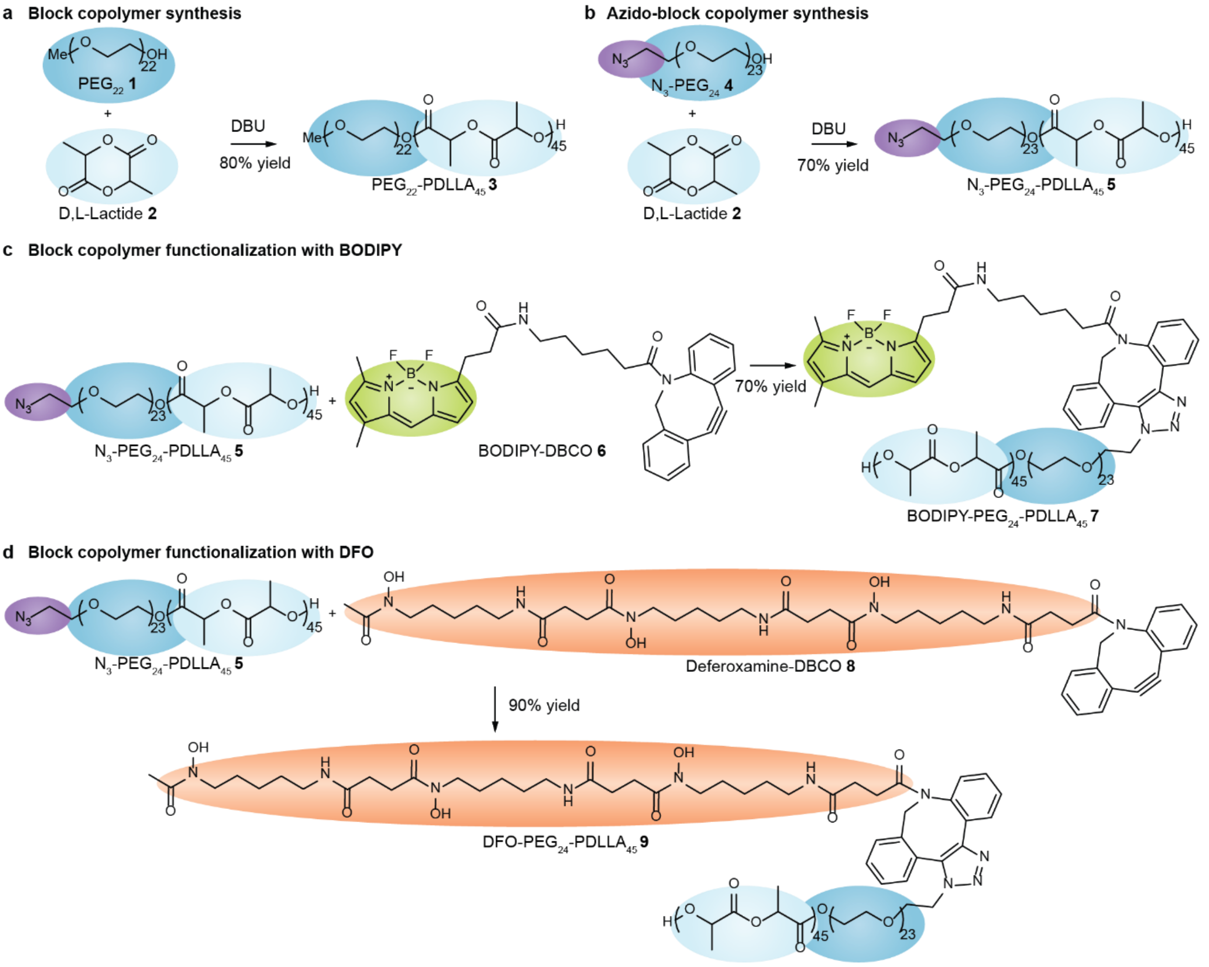

Supplementary Fig. 1 │Synthetic scheme of polymers and conjugates. Reagents and conditions: **a**, DBU, DCM, 25°C, Argon, 2 hours, 80%. **b**, DBU, DCM, 25°C, Argon, 2 hours, 70%. **c**, DCM, 25°C, Argon, 24 hours, 70%. **d**, DMSO, 50°C, Argon, 24 hours, 90%.

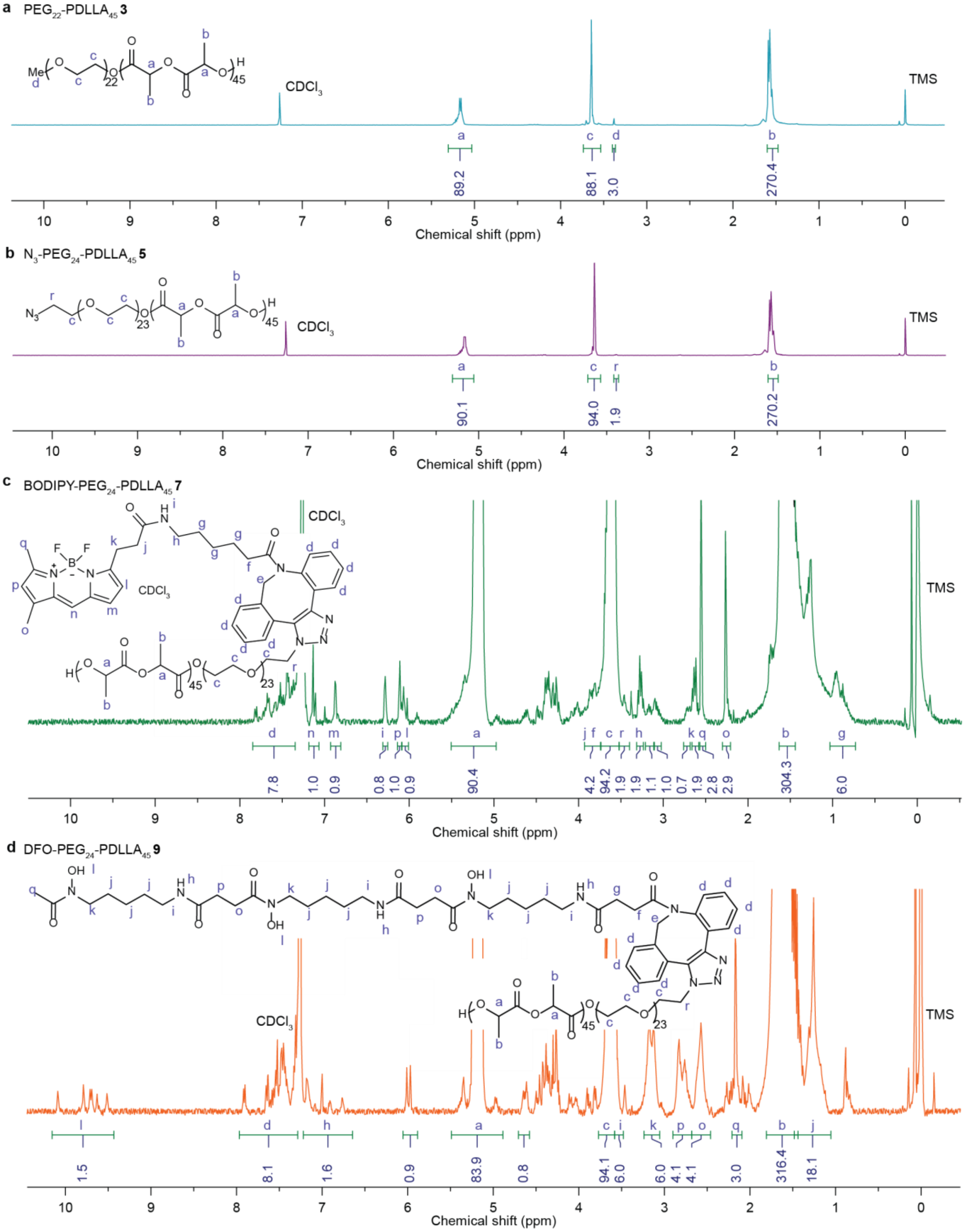

Supplementary Fig. 2 │^{1}H NMR spectra of PEG-PDLLA block copolymers and polymer-conjugates. ^{1}H NMR spectra ($CDCl_3$ with v/v 0.05% TMS, 400 mHz) of **a**, PEG_{22}-$PDLLA_{45}$ **3**. **b**, N_3-PEG_{24}-$PDLLA_{45}$ **5**. **c**, BODIPY-PEG_{24}-$PDLLA_{45}$ **7**. **d**, DFO-PEG_{24}-$PDLLA_{45}$ **9**.

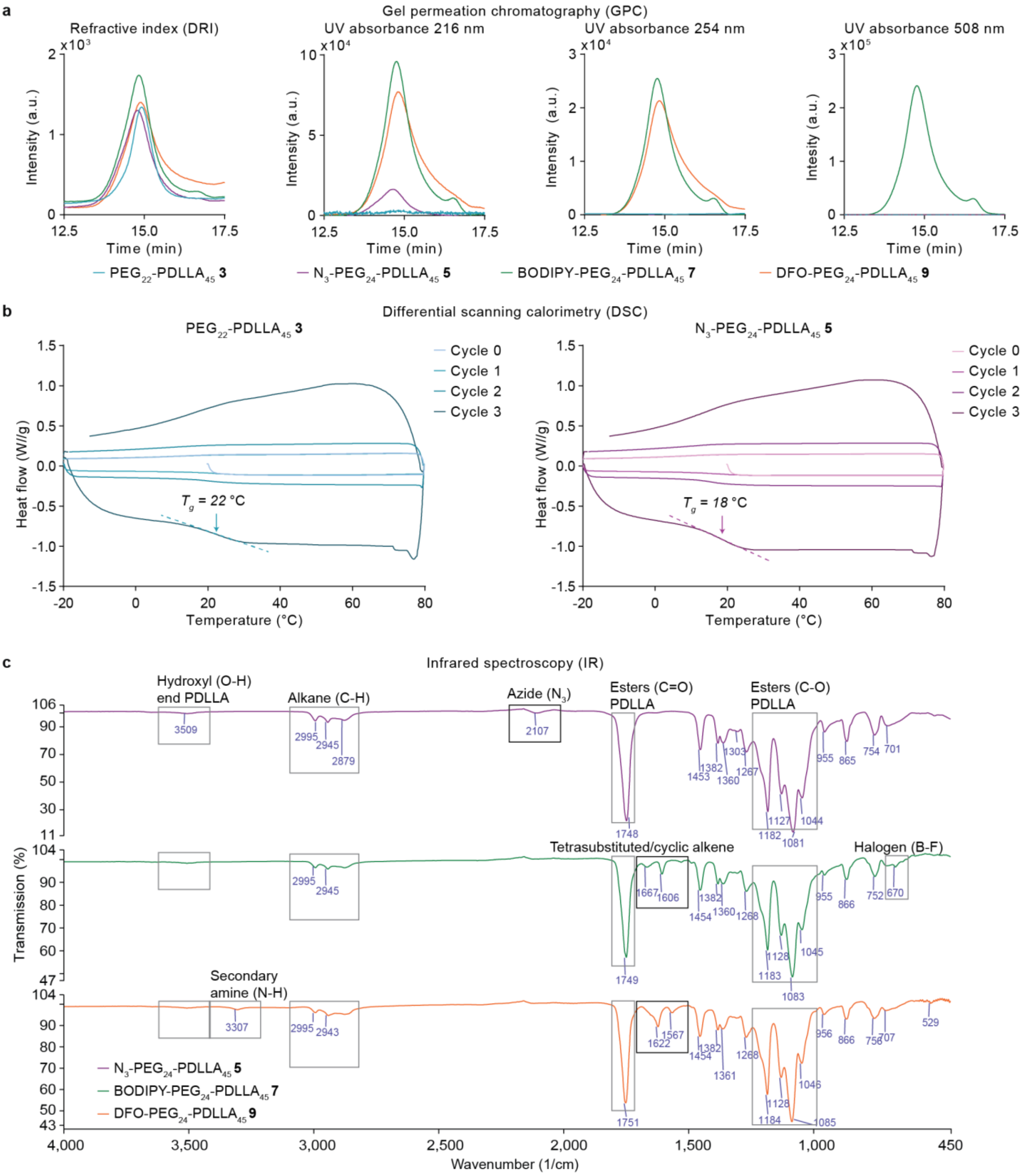

Supplementary Fig. 3 │Characterization of block copolymers and polymer-conjugates with GPC, DSC, and IR. a, Gel permeation chromatography (GPC) refractive index (RI) and UV absorbance signals (RI) of polymers **3**, **5**, **7**, **9**. Signals overlap with RI signals at wavelengths that are characteristic for the functional groups. **b**, Differential scanning calorimetry (DSC) results of polymers **3** and **5**, showing their glass transition temperature (T_g). **c**, Infrared spectroscopy (IR) spectra of polymers **5**, **7**, **9**.

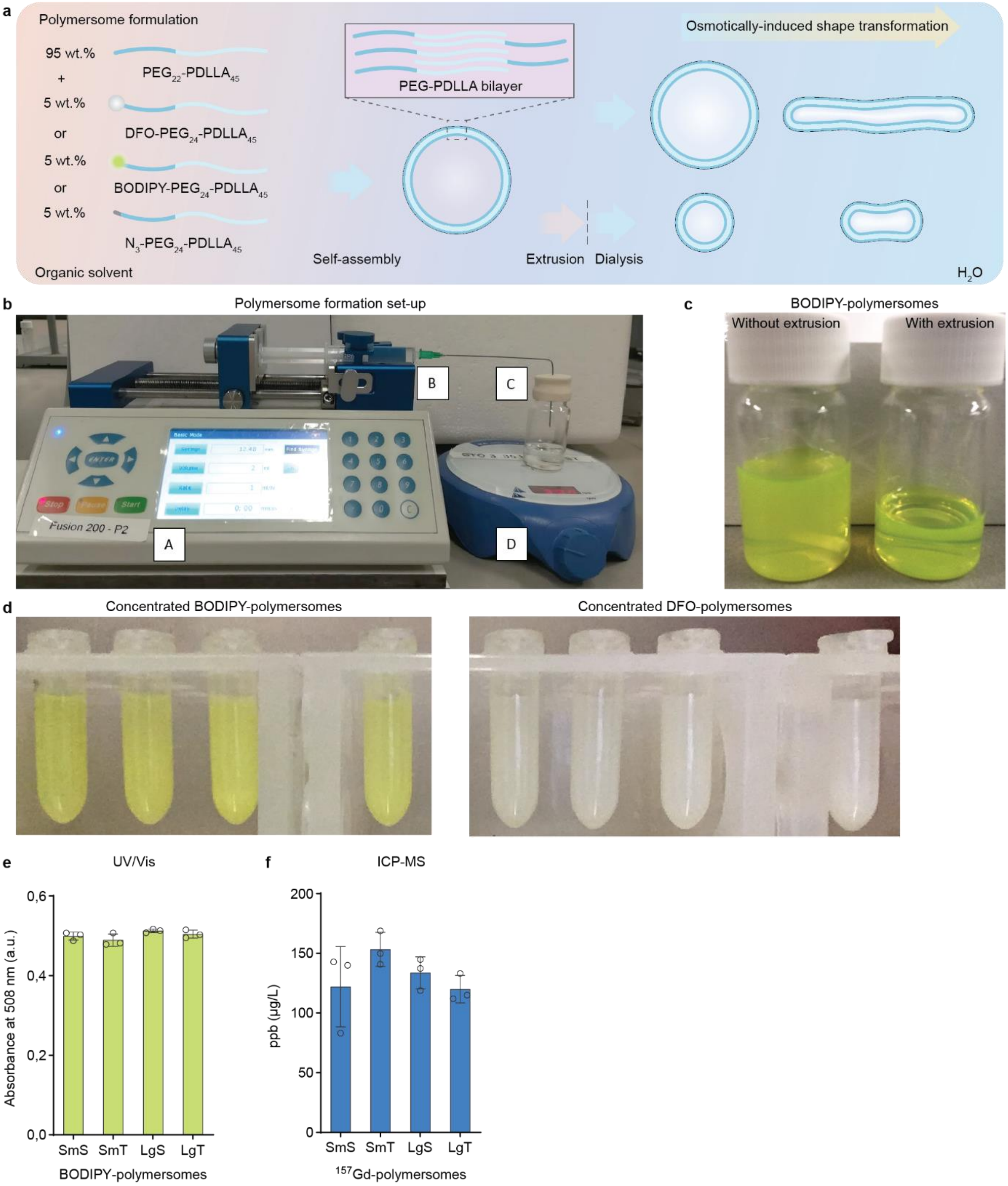

Supplementary Fig. 4 │Polymersome formulation. a, Schematic representation of the polymersome formulation procedure used to generate DFO- or BODIPY-labeled polymersomes. Polymersome vesicles self-assemble when water is added to a 95 wt.% PEG_{22}-$PDLLA_{45}$ **3** solution with either 5 wt.% DFO-PEG_{24}-$PDLLA_{45}$ **7** or 5 wt.% BODIPY-PEG_{24}-$PDLLA_{45}$ **9** in organic solvent. Small polymersomes were formed by performing extrusion prior to dialysis. Dialysis against water leads to the formation of large or small spheres, whereas dialysis against salt solution leads to their osmotically-induced shape transformation into large or small tubes. **b**, The set-up utilized to induce PEG-PDLLA block copolymers to self-assemble into polymersomes via the solvent switch methodology: A fusion syringe pump (A) is used to add water (2 mL (without extrusion) or 1 mL (with extrusion) at a rate of 1 mL/hour) (B) to the polymer solution – 20 mg in a 2 mL mixture of THF and dioxane (4:1 v/v) - (C) while stirring (D). **c**, Large (5 mg/mL) and small (6.6 mg/mL) BODIPY-polymersomes before dialysis. **d**, BODIPY- and DFO-polymersomes after dialysis and concentration (10 mg/mL). **e**, UV/Vis analyses reveal similar BODIPY-polymersome absorbance (10 mg/mL polymer) at λ = 508 nm (n=3), indicating equal labeling that allows BODIPY signal comparison by flow cytometry. **f**, ICP-MS analyses reveal similar ^{157}Gd-polymersome labeling (2.5 mg/mL polymer) (n=3), indicating equal labeling that allows ^{157}Gd signal comparison by mass cytometry. Data are presented as mean ± SD.

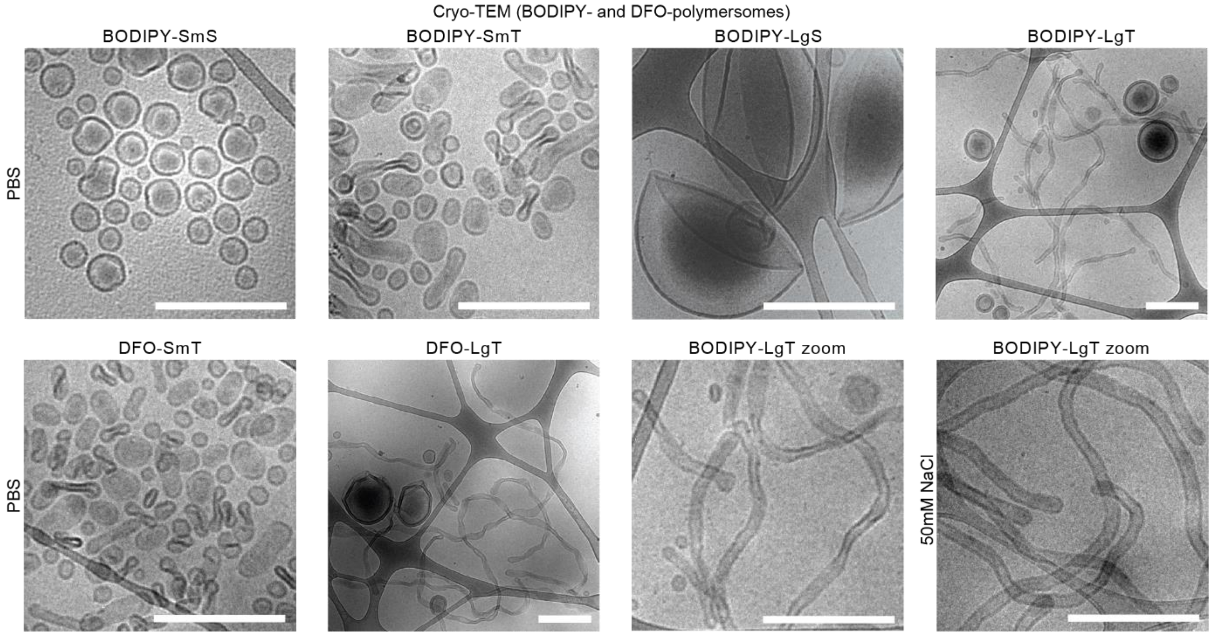

Supplementary Fig. 5 │Cryo-TEM images of BODIPY- and DFO-polymersomes. Cryo-TEM micrographs of polymersomes in PBS and zoom-ins of BODIPY-LgT in PBS and 50 mM NaCl. Similar results for polymersome topologies were obtained in three independent experiments. Scale bar = 500 nm. Cryo-TEM = cryogenic transmission electron microscopy, SmS = small spheres, SmT = small tubes, LgS = large spheres, LgT = large tubes, PBS = phosphate buffered saline.

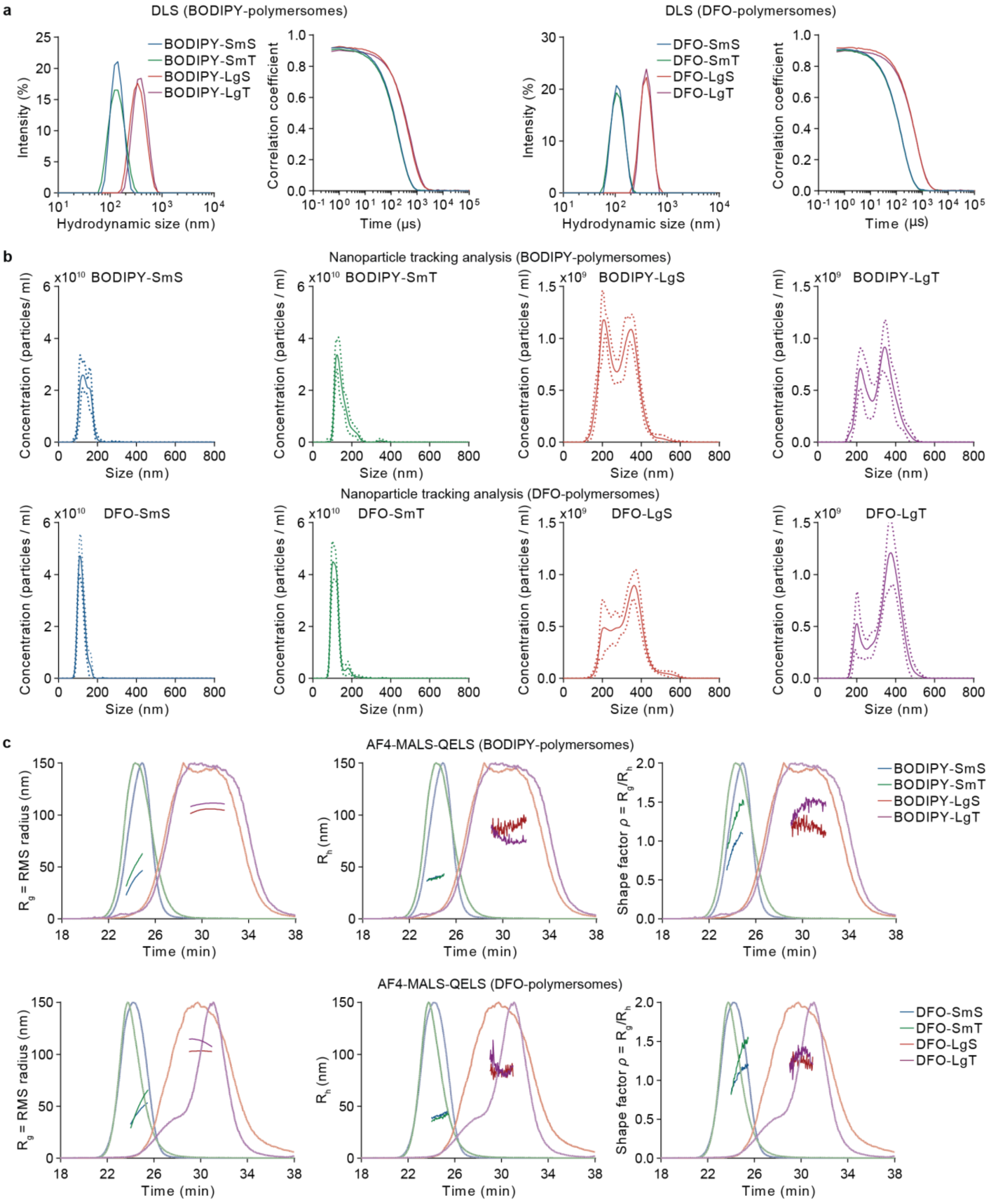

Supplementary Fig. 6 │Light scattering analysis of BODIPY- and DFO-polymersomes. a, Dynamic light scattering (DLS) intensity profiles and correlation functions (n=3). **b**, Nanoparticle tracking analysis (NTA) data (n=5). **c**, Asymmetric flow-field flow fractionation (AF4) fractograms with light scattering (MALS-QELS) analyses results of the polymersomes in PBS at 33°C. Scattering profiles are relatively scaled. Insets indicate radii of gyration (R_g = Root Mean Square (RMS) radius), hydrodynamic radii (R_h), and their ratio. R_g and R_h were determined by MALS and QELS, respectively; comparing both radii results in the respective shape factor ρ (defined as R_g/R_h), which provides quantitative information on particle shape. Data are presented as mean in **a** and mean ± SD in **b**. SmS = small spheres, SmT = small tubes, LgS = large spheres, LgT = large tubes.

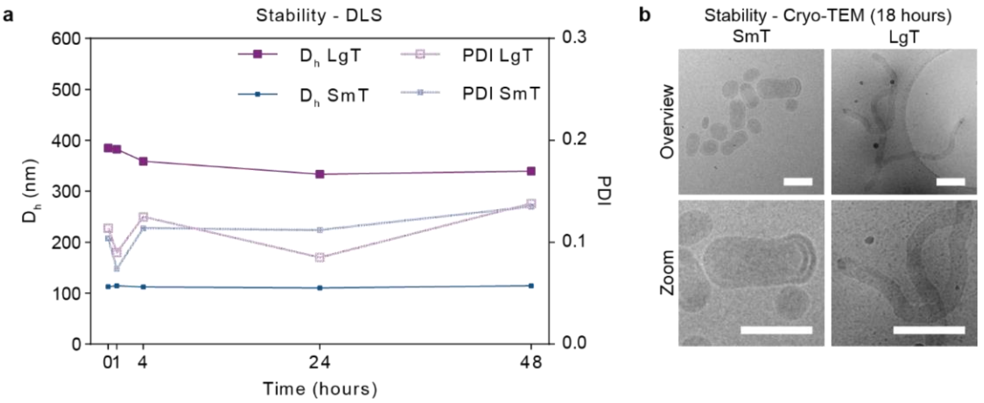

Supplementary Fig. 7 │Polymersome stability analysis. a, Stability analyses using DLS for large tubes (LgT) and small tubes (SmT) under physiological conditions (n=3). Hydrodynamic diameters (D_h) and polydispersity indices (PDI) were measured after incubating the polymersomes for 0, 1, 4, 24, and 48 hours in cell culture medium supplemented with serum (LgT 1.25 mg/mL polymer, 5.0 % FCS and SmT 2.50 mg/mL polymer, 7.5% FCS, 37°C, 500 rpm). D_h and PDI do not significantly change after incubation. **b**, Stability analyses using cryo-TEM of large tubes (LgT) and small tubes (SmT) under physiological conditions. Cryo-TEM micrographs acquired after 18 hours of incubation, as described in (**a**), show that the polymersomes retained their tubular morphology. Similar results for polymersome topologies were obtained in three independent experiments. Scale bar = 200 nm. Data are presented as mean.

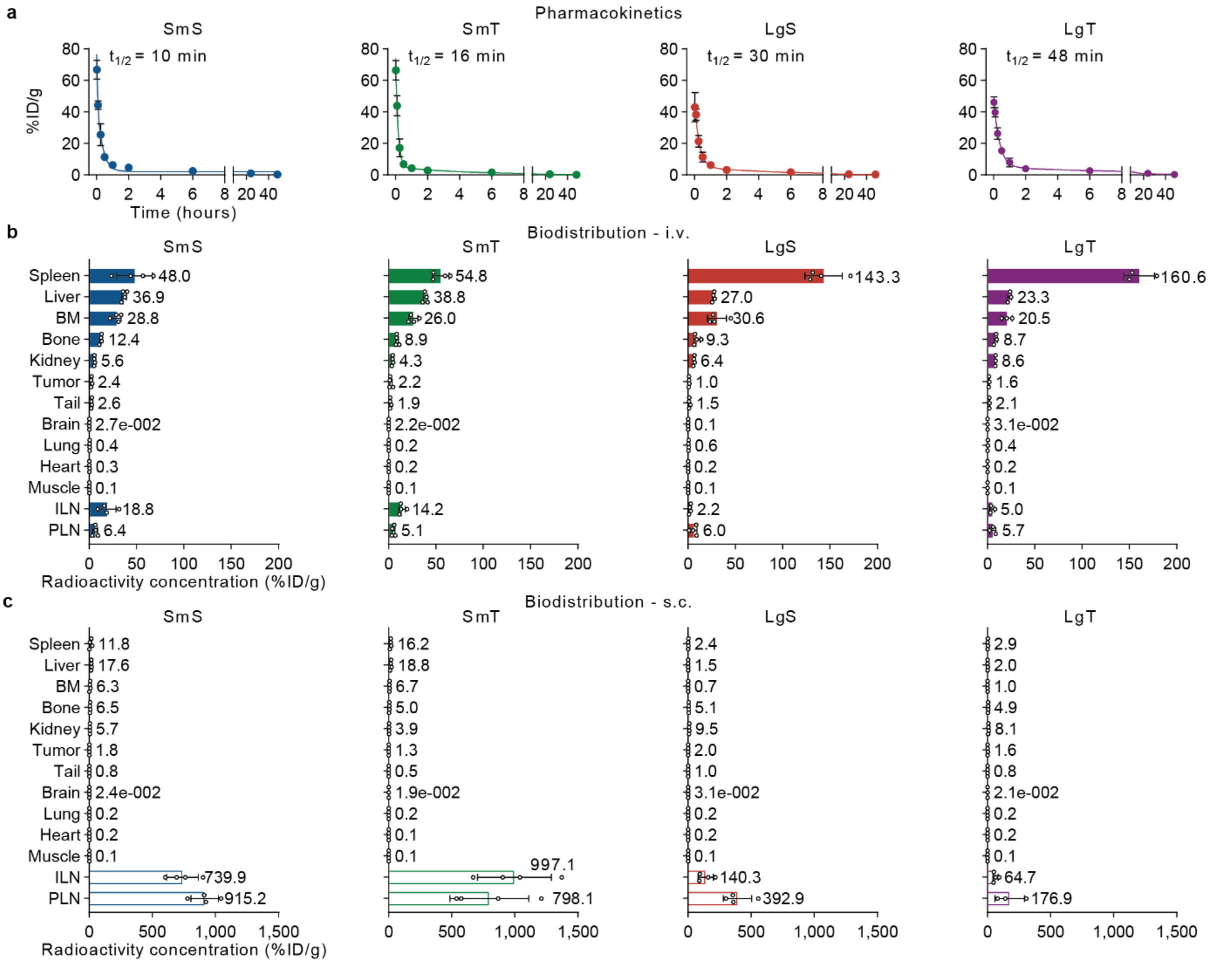

Supplementary Fig. 8 │Pharmacokinetics and biodistribution of ^{89}Zr-polymersomes in B16F10 melanoma-bearing C57BL/6 mice. a, Blood half-life time ($t_{1/2}$) of ^{89}Zr-polymersomes with various topologies upon i.v. injection, n=4/group. **b**, Biodistribution of i.v. administered ^{89}Zr-polymersomes after 48 hours, as determined by *ex vivo* gamma counting, n=4/group. **c**, Biodistribution of s.c. administered ^{89}Zr-polymersomes after 48 hours, as measured by *ex vivo* gamma counting, n=4/group. All data are presented as mean ± SD. i.v. = intravenously via lateral tail vein injection, s.c. = subcutaneously via footpad injection, SmS = small spheres, SmT = small tubes, LgS = large spheres, LgT = large tubes, BM = bone marrow, ILN = iliac lymph node, PLN = popliteal lymph node, %ID/g = percentage injected dose per gram of tissue.

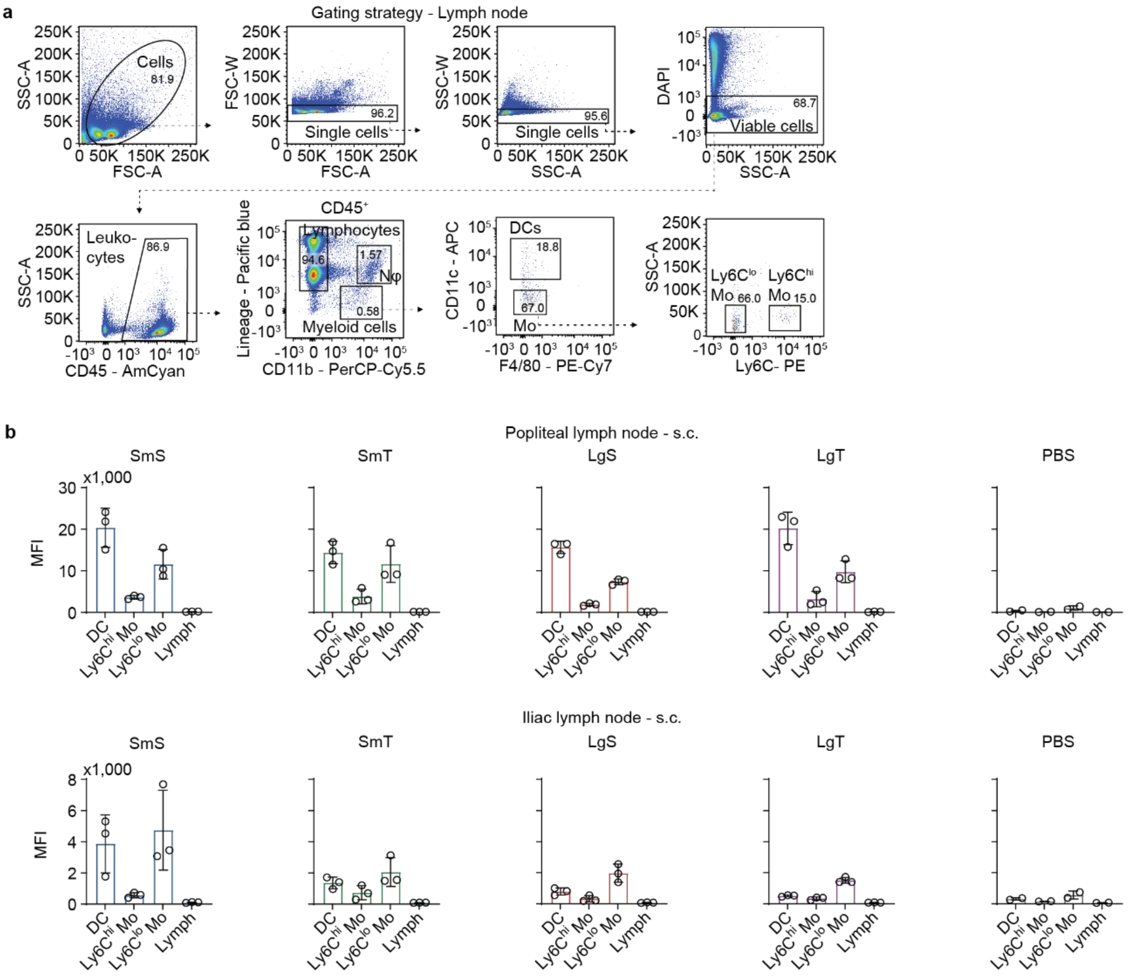

Supplementary Fig. 9 │Immune cell specificity of BODIPY-polymersomes in lymph nodes of B16F10 melanoma-bearing C57BL/6 mice. a, Representative flow cytometry plots of lymph nodes showing the gating strategy to identify dendritic cells (DCs), Ly6C^{hi} and Ly6C^{lo} monocytes (Mo), and lymphocytes 48 hours after s.c. administration of BODIPY-polymersomes in B16F10 melanoma-bearing C57BL/6 mice. **b**, Uptake of s.c. administered BODIPY-polymersomes in specific immune cell types in the popliteal and iliac lymph nodes after 48 hours. The average BODIPY mean fluorescent intensity (MFI) for each cell type as assessed by flow cytometry, n=3/group. PBS represents a control group to indicate background MFI levels, n=2/group. All data are presented as mean ± SD. s.c. = subcutaneously via footpad injection, SmS = small spheres, SmT = small tubes, LgS = large spheres, LgT = large tubes, PBS = phosphate buffered saline, DC = dendritic cells, Mo = monocytes.

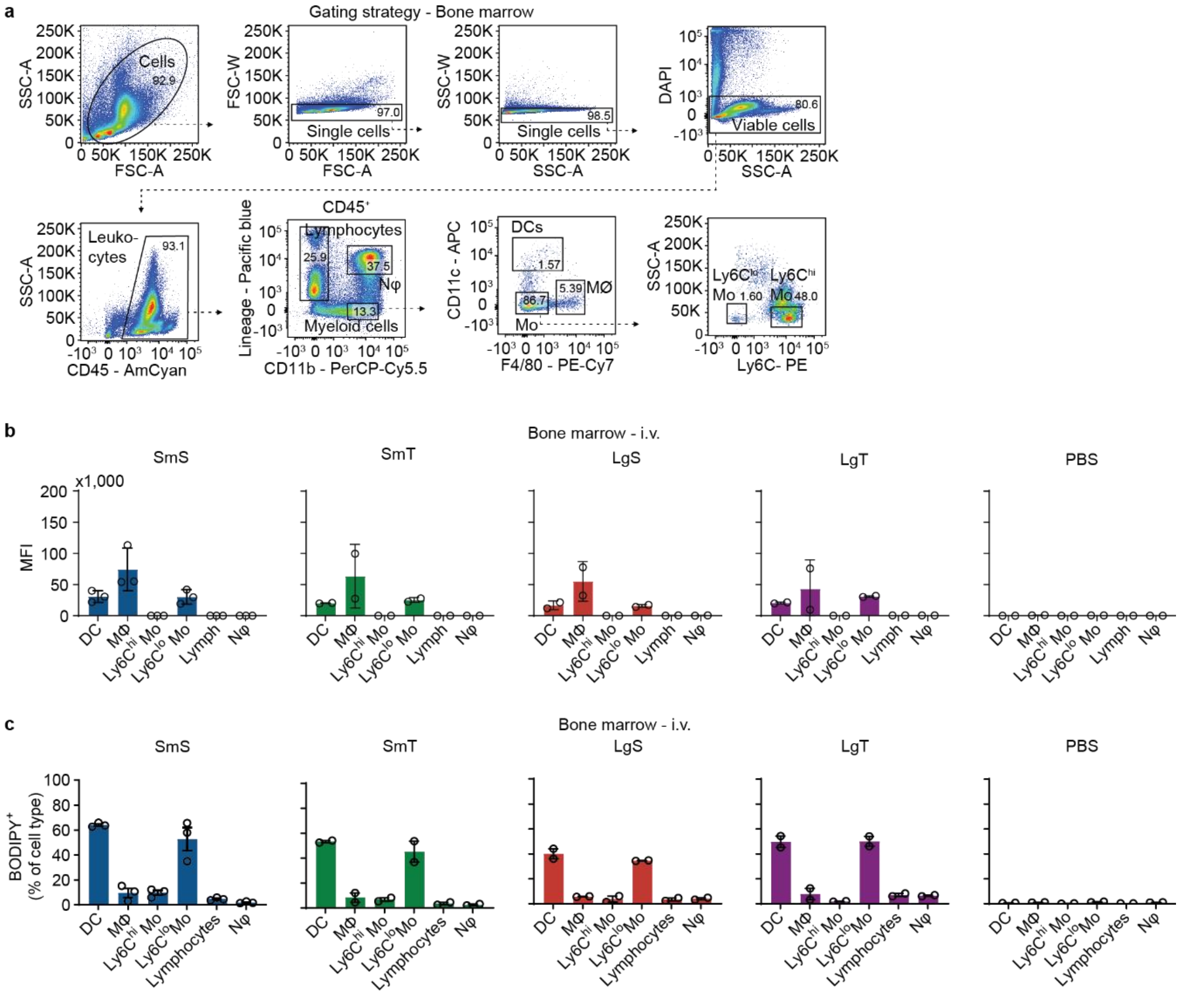

Supplementary Fig. 10 │Immune cell specificity of BODIPY-polymersomes in bone marrow of B16F10 melanoma-bearing C57BL/6 mice. a, Representative flow cytometry plots of bone marrow showing the gating strategy to identify dendritic cells (DCs), Ly6C^{hi} and Ly6C^{lo} monocytes (Mo), lymphocytes, neutrophils (Nφ), and macrophages (MΦ) 48 hours after i.v. administration of BODIPY-polymersomes in B16F10 melanoma-bearing C57BL/6 mice. **b** and **c**, Uptake of i.v. administered BODIPY-polymersomes by immune cells in the bone marrow after 48 hours, n=3/group for SmS and n=2/group for other conditions. Bar graphs of (**b**) show the average BODIPY mean fluorescent intensity (MFI) for each cell type and bar graphs of (**c**) show the average percentage of BODIPY+ cells within each cell type, both assessed by flow cytometry. PBS represents a control group to indicate background MFI levels and to perform gating of BODIPY+ cells, respectively. All data are presented as mean ± SD. i.v. = intravenously via lateral tail vein injection, SmS = small spheres, SmT = small tubes, LgS = large spheres, LgT = large tubes, PBS = phosphate buffered saline, DC = dendritic cells, MΦ = macrophages, Mo = monocytes, Nφ = neutrophils.

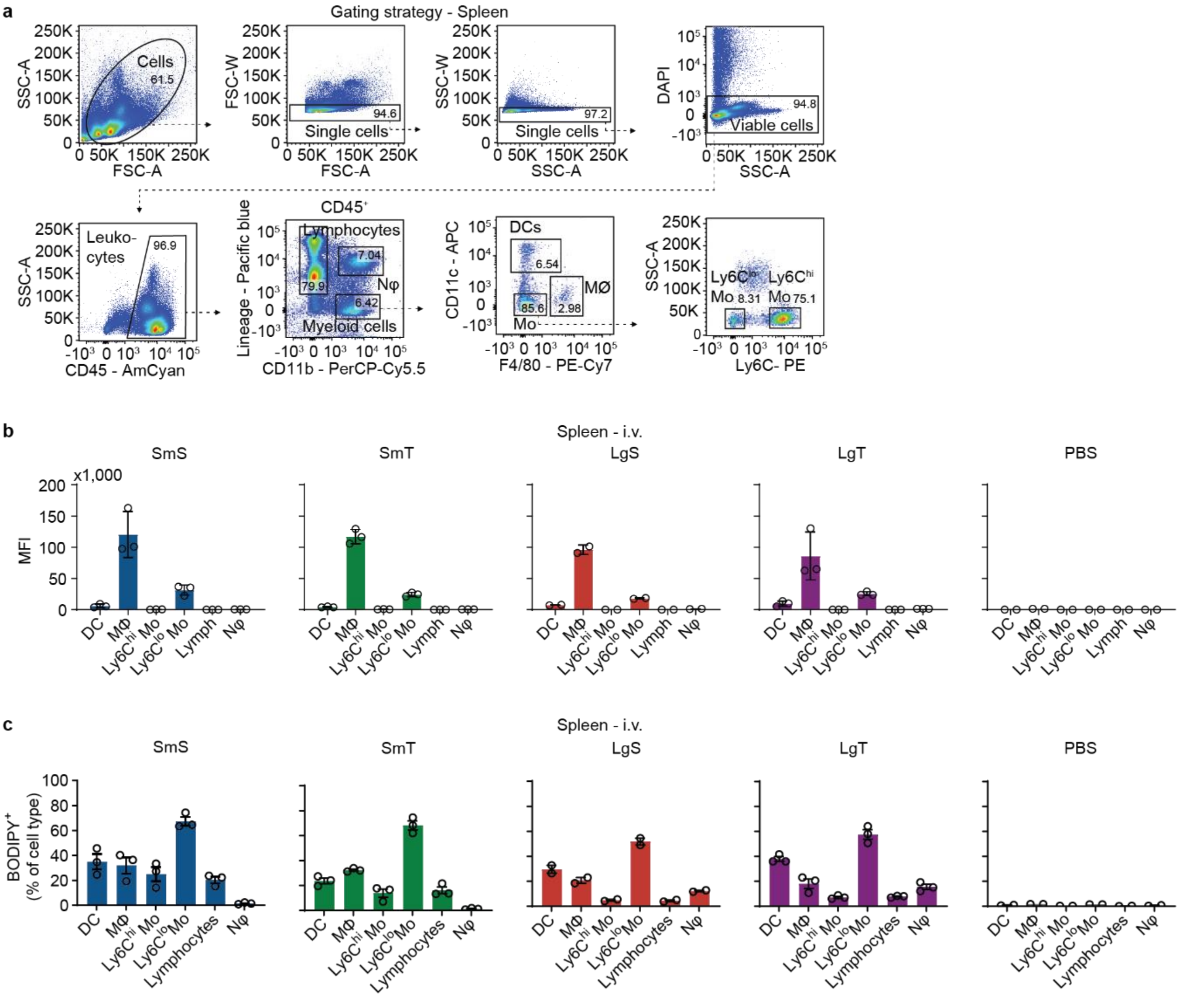

Supplementary Fig. 11 │Immune cell specificity of BODIPY-polymersomes in spleen of B16F10 melanoma-bearing C57BL/6 mice. a, Representative flow cytometry plots of spleen showing the gating strategy to identify dendritic cells (DCs), Ly6C^{hi} and Ly6C^{lo} monocytes (Mo), lymphocytes, neutrophils (Nφ), and macrophages (MΦ) 48 hours after i.v. administration of BODIPY-polymersomes in B16F10 melanoma-bearing C57BL/6 mice. **b** and **c**, Uptake of i.v. administered BODIPY-polymersomes by immune cells in the spleen after 48 hours, n=3/group for SmS, SmT and LgS, and n=2/group for LgS and PBS. Bar graphs of (**b**) show the average BODIPY mean fluorescent intensity (MFI) for each cell type and bar graphs of (**c**) show the average percentage of BODIPY+ cells within each cell type, both assessed by flow cytometry. PBS represents a control group to indicate background MFI levels and to perform gating of BODIPY+ cells, respectively. All data are presented as mean ± SD. i.v. = intravenously via lateral tail vein injection, SmS = small spheres, SmT = small tubes, LgS = large spheres, LgT = large tubes, PBS = phosphate buffered saline, DC = dendritic cells, MΦ = macrophages, Mo = monocytes, Nφ = neutrophils.

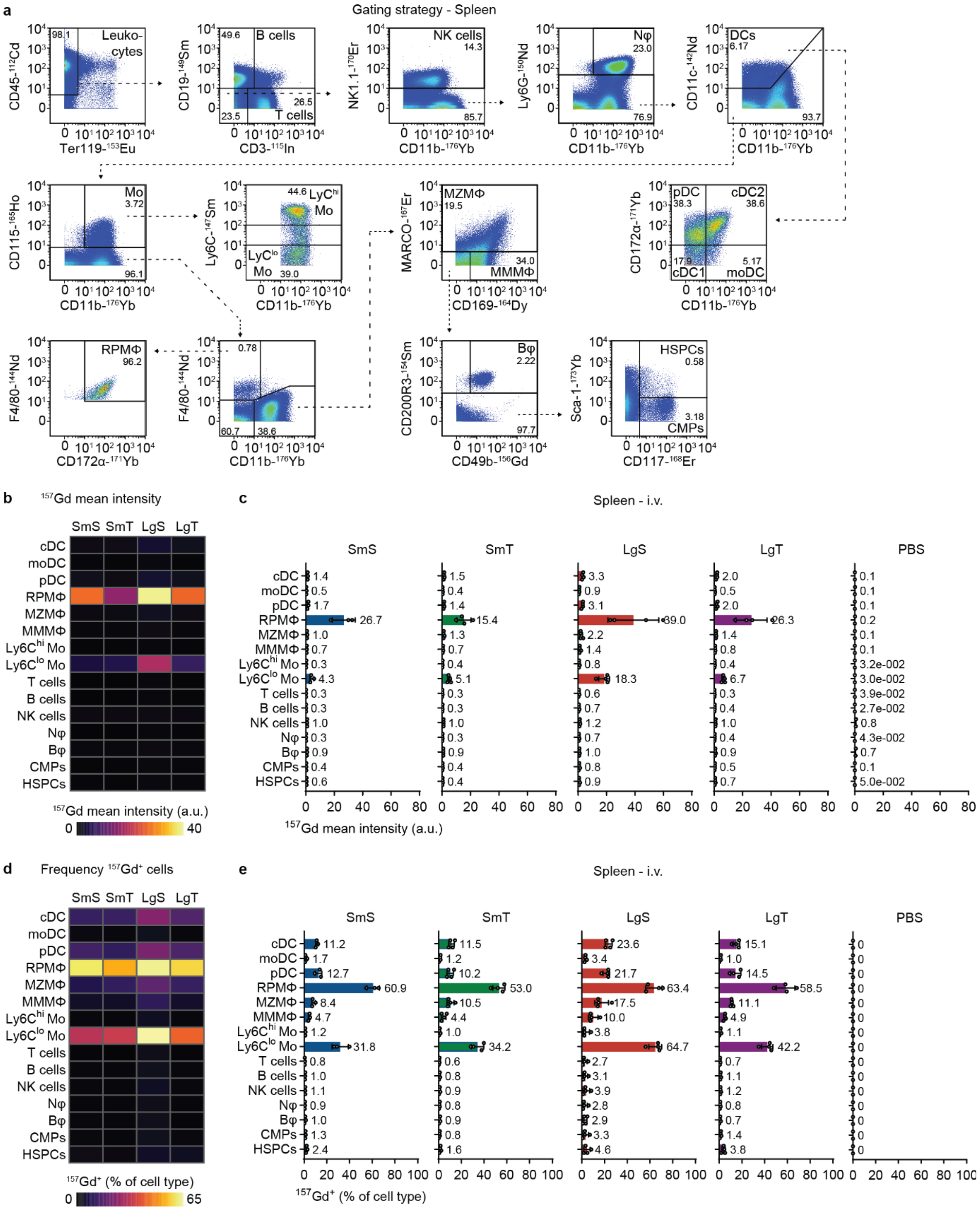

Supplementary Fig. 12 │ Immune cell specificity of ^{157}Gd-polymersomes in spleens of B16F10 melanoma-bearing C57BL/6 mice. a, Representative mass cytometry plots of spleen, showing the gating strategy to identify specific immune cell types 48 hours after i.v. administration of ^{157}Gd-polymersomes in B16F10 melanoma-bearing C57BL/6 mice. **b-e**, Uptake of i.v. administered ^{157}Gd-polymersomes by specific immune cells in the spleen, n=3 for SmS and n=4/group for all other conditions. Heatmap of (**b**) and bar graphs of (**c**) show ^{157}Gd mean intensity for each cell type and heatmap of (**d**) and bar graphs of (**e**) show the average percentage of $^{157}Gd^{+}$ cells within each cell type, both assessed by mass cytometry. PBS represents a control group (n=2) to indicate background mean intensity levels and to perform gating of $^{157}Gd^{+}$ cells, respectively. All data are presented as mean ± SD. NK cells = natural killer cells, Nφ = neutrophils, cDC = classical dendritic cells, moDCs = monocyte-derived dendritic cells, pDC = plasmacytoid dendritic cells, Mo = monocytes, RPMΦ = red pulp macrophages, MZMΦ = marginal zone macrophages, MMMΦ = marginal metallophilic macrophages, Bφ = basophils, HSPC = hematopoietic stem and progenitor cells, CMP = common myeloid

progenitors, i.v. = intravenously via lateral tail vein injection, SmS = small spheres, SmT = small tubes, LgS = large spheres, LgT = large tubes.

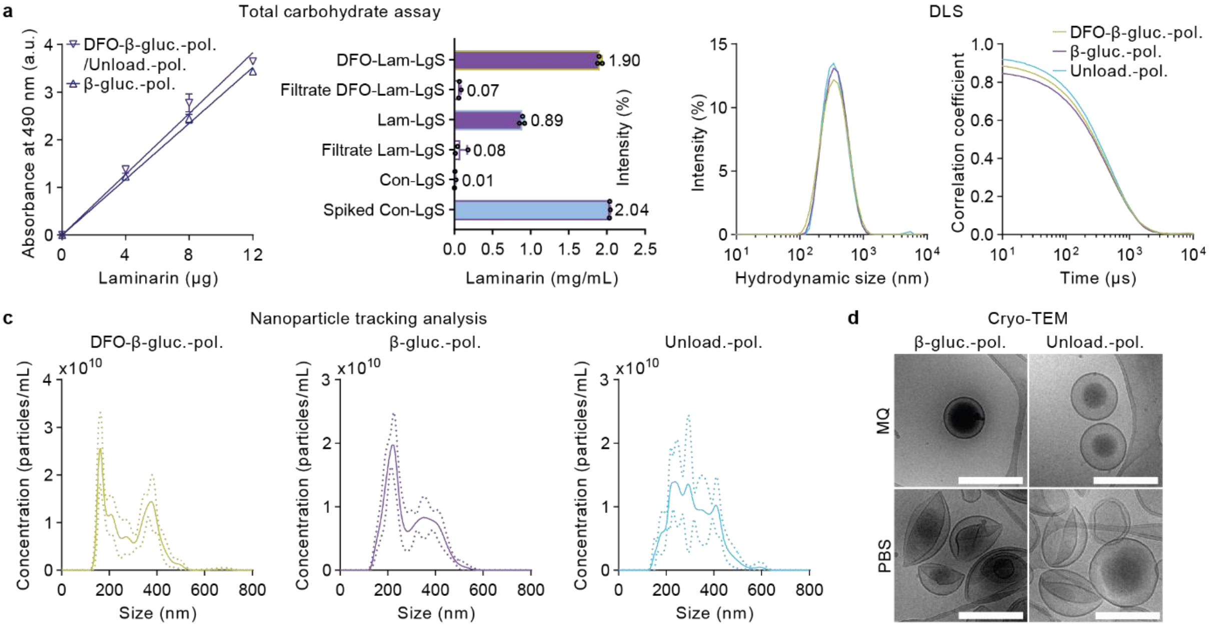

Supplementary Fig. 13 │Characterization of β-glucan-loaded and unloaded polymersomes. a, β-glucan (laminarin) concentration quantification using a standard colorimetric carbohydrate assay. (Left) Standard curves used for quantification. (Right) The determined laminarin concentrations and encapsulation efficiencies (ee%) for DFO-β-glucan-polymersomes (DFO-β-gluc.pol.) and β-glucan-polymersomes (β-gluc.pol.). Samples' filtrate (Filtr., from the concentration steps) and unloaded polymersomes (unload.-pol.) were used as negative controls, and laminarin-spiked unloaded polymersomes was used as a positive control, n=3/group. **b**, Dynamic light scattering (DLS) intensity profiles and correlation functions (n=3). **c**, Nanoparticle tracking analysis (NTA) data (n=5). **d**, Cryo-TEM images of β-glucan-polymersomes and unloaded polymersomes in Milli-Q water and PBS. Similar results for polymersome topologies were obtained in three independent experiments. Scale bar = 500 nm. All data are represented as mean ± SD.

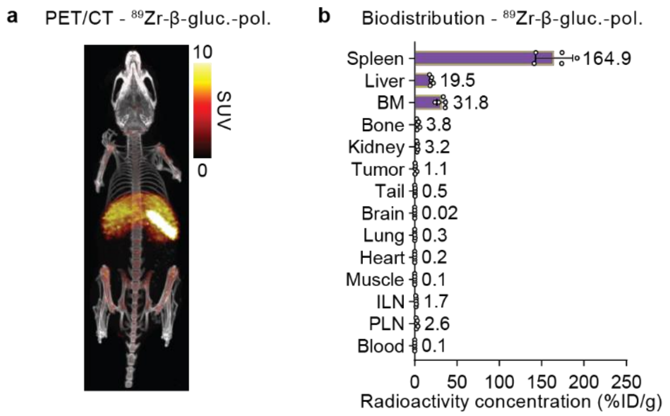

Supplementary Fig. 14 │Biodistribution of ^{89}Zr-β-glucan-polymersomes in B16F10 melanoma-bearing C57BL/6 mice. a, Representative whole-body PET image 48 hours after i.v. administration of ^{89}Zr-β-glucan-polymersomes. **b**, Biodistribution of i.v. administered ^{89}Zr-β-glucan-polymersomes as determined by *ex vivo* gamma counting after 48 hours, n=5/group. In agreement with previous observations for ^{89}Zr-LgS, ^{89}Zr-β-glucan-polymersomes strongly accumulate in the spleen. Data are presented as mean ± SD. BM = bone marrow, ILN = iliac lymph node, PLN = popliteal lymph node, %ID/g = percentage injected dose per gram of tissue.

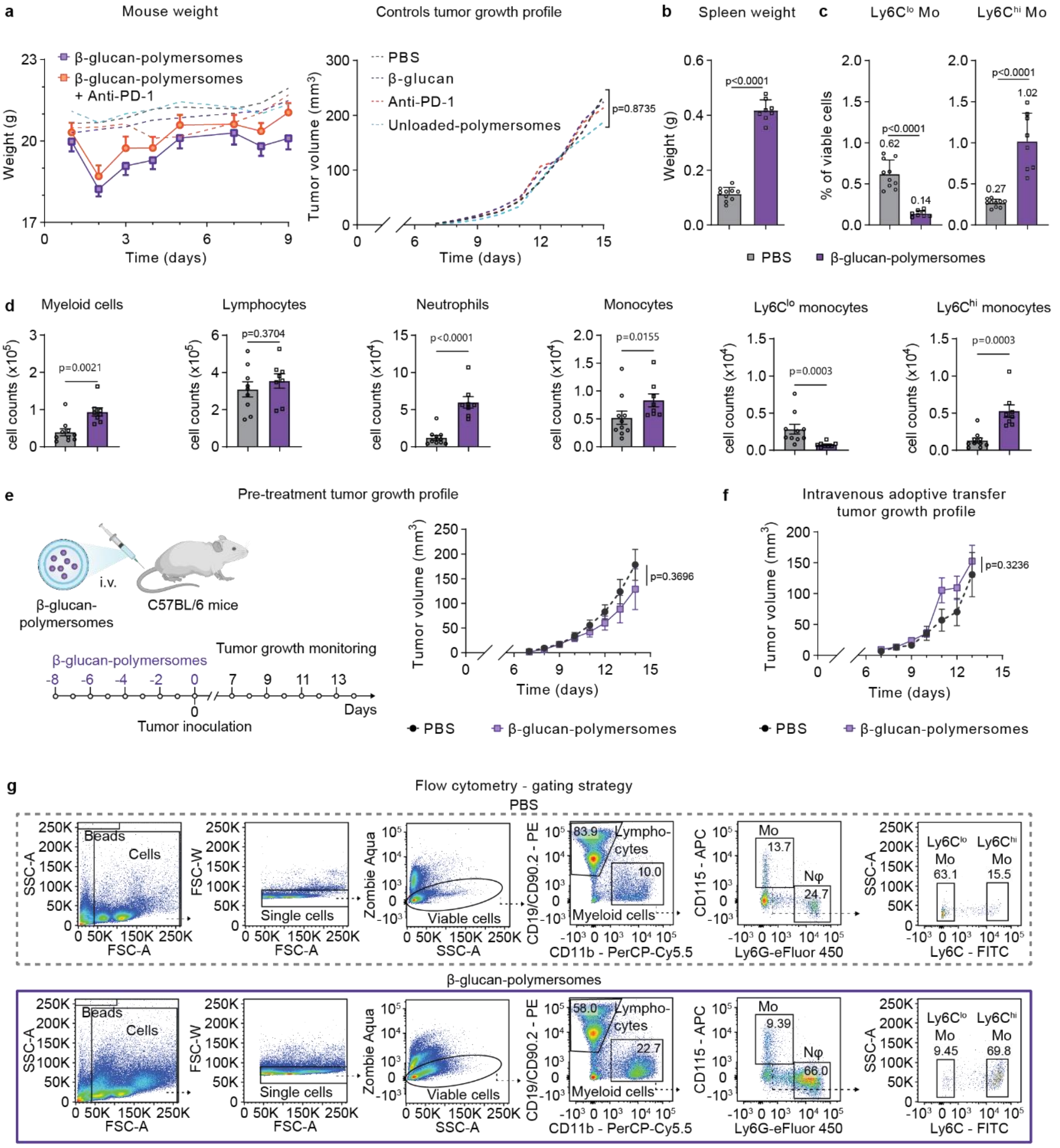

Supplementary Fig. 15 │*In vivo* immunotherapeutic application of β-glucan-polymersomes. **a**, β-glucan, anti-PD-1, unloaded polymersomes or β-glucan-polymersomes, either as a monotherapy, or combined with anti-PD-1 checkpoint inhibition, were administered i.v. to B16F10-melanoma-bearing C57BL/6 mice. Mouse weight and tumor growth were monitored for 8 days. The *in vivo* tumor growth profiles shows that β-glucan and anti-PD-1 did not reduce tumor size or growth rate, as compared to PBS or unloaded polymersome-treated control groups, n=10/group. **b**, Spleen weight of B16F10-melanoma-bearing C57BL/6 mice treated with β-glucan-polymersomes (n=8) is approximately four-fold increased compared to PBS controls (n=10). **c**, Relative abundance of splenic Ly6C^{lo} monocytes, and Ly6C^{hi} monocytes in B16F10-melanoma-bearing C57BL/6 mice treated with β-glucan-polymersomes (n=8) and PBS (n=10). **d,** Absolute abundance per whole spleen of myeloid cells, lymphocytes, neutrophils, monocytes, Ly6C^{lo} monocytes, and Ly6C^{hi} monocytes in B16F10-melanoma-bearing C57BL/6 mice treated with β-glucan-polymersomes (n=8) and PBS (n=10). **e**, Healthy C57BL/6 mice were treated with i.v. administration of β-glucan-polymersomes and tumor growth was monitored for 8 days. The *in vivo* tumor growth profiles show that prophylactic administration of β-glucan-polymersomes did not reduce tumor size or growth rate, as compared to the PBS-pretreated control group, n=10/group. **f**, Healthy C57BL/6 mice were treated i.v. with β-glucan-polymersomes and splenic myeloid cells were isolated and administrated i.v. to B16F10-bearing C57BL/6 mice. Tumor growth was monitored for 8 days. The *in vivo* tumor growth profiles show that β-glucan-polymersome-treated myeloid cells did not reduce tumor size or growth rate in an adoptive transfer setting, n=10/group. **g**, Representative flow cytometry plots of

splenic leukocytes harvested from B16F10 melanoma-bearing C57BL/6 mice treated with PBS (top) and β-glucan-polymersomes (bottom). Data are presented as mean ± SE. P values were calculated using a two-way ANOVA to compare tumor growth or an unpaired Mann-Whitney test to compare spleen weight and splenic immune cells. All P values are two-tailed and $P<0.05$ is considered significant. PBS = phosphate buffered saline, anti-PD-1 = anti-programmed cell death protein 1, i.v. = intravenously via lateral tail vein injection, Mo = monocytes, Nφ = neutrophils. Schematic illustrations of mouse and syringe were created with BioRender.com.

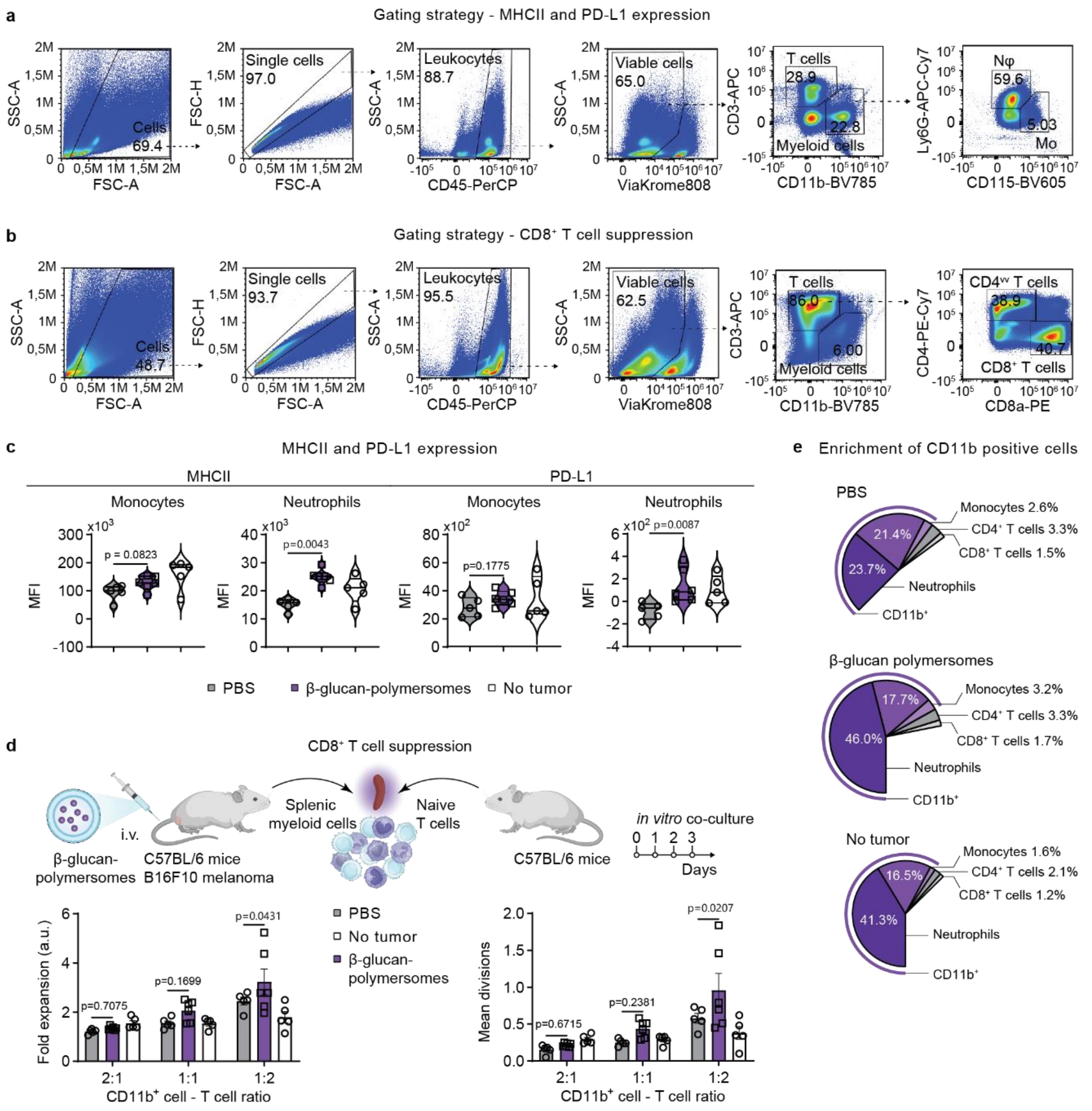

Supplementary Fig. 16 │ Immunosuppressive capacity of splenic neutrophils and monocytes after β-glucan-polymersome treatment. a, Representative flow cytometry plots showing the gating strategy to identify monocytes and neutrophils (Nφ) from the spleen of B16F10 melanoma-bearing C57BL/6 mice. **b**, Representative flow cytometry plots showing the gating strategy to identify CD8$^+$ T cells from *in vitro* co-cultures with splenic CD11b$^+$ myeloid cells. **c**, β-glucan-polymersomes were administered to B16F10 melanoma-bearing C57BL/6 mice to determine the expression of MHCII and PD-L1 on monocytes and neutrophils. The average mean fluorescent intensity for each cell type as assessed by flow cytometry, n=5/group for PBS and no tumor, and n=6/ for β-glucan-polymersomes. The no tumor group represents a control group to indicate physiological expression levels. **d**, β-glucan-polymersomes were administered to B16F10 melanoma-bearing C57BL/6 mice. Splenic CD11b$^+$ myeloid cells from treated mice and T cells from naïve mice were isolated and co-cultured for three days in the presence of Dynabeads™ (1:1) and recombinant IL-2 (50IU/mL). CD8$^+$ T cell suppression measured as the average fold expansion and number of divisions as assessed by flow cytometry, n=5/group for PBS and no tumor, and n=6/ for β-glucan-polymersomes. The no tumor group represents a control group to indicate physiological T cell proliferation. **e,** Relative abundance of splenic myeloid cells (CD11b$^+$), neutrophils, monocytes, CD4$^+$ T cells, and CD8$^+$ T cells after CD11b positive cell enrichment with CD11b magnetic beads, n=5-6/group. Expression data are presented as violin plots and proliferation data are presented as mean ± SE. P values were calculated for the β-glucan-polymersome and PBS-treated groups using an unpaired Mann-Whitney test to compare expression levels or a two-way ANOVA to compare CD8$^+$ T cell suppression. All P values are two-tailed and P<0.05 is considered significant. Mo = monocytes, Nφ = neutrophils, MHCII = major histocompatibility complex II, PD-L1 = programmed death-ligand 1, i.v. = intravenously via lateral tail vein injection. Schematic illustrations of mice, spleens, syringes, and cells were created with BioRender.com.

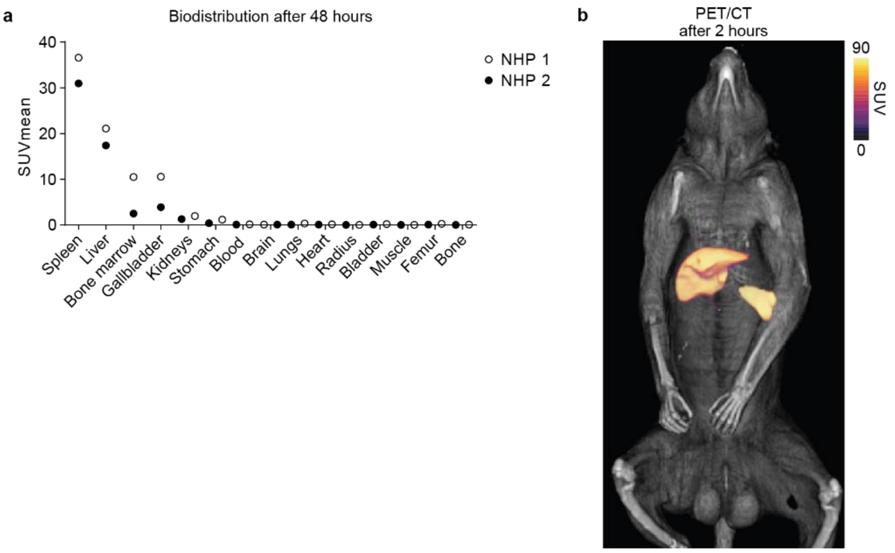

Supplementary Fig. 17 │Biodistribution of ^{89}Zr-β-glucan-polymersomes in non-human primates. ^{89}Zr-β-glucan-polymersomes were i.v. injected in two NHPs, followed by PET/CT imaging. **a**, Biodistribution of ^{89}Zr-β-glucan-polymersomes in two NHPs 48 hours after administration. **b**, PET/CT images at 2 hours display accumulation in the spleen and liver. NHP = non-human primate, SUV = standardized uptake value.

References

1. Wauters, A. C. *et al.* Development of Morphologically Discrete PEG-PDLLA Nanotubes for Precision Nanomedicine. *Biomacromolecules* **20**, 177–183 (2019).
2. Barge, A., Cravotto, G., Gianolio, E. & Fedeli, F. How to determine free Gd and free ligand in solution of Gd chelates. A technical note. *Contrast Media Mol. Imaging* **1**, 184–188 (2006).
