# Supplementary material for: Polymersomes with splenic avidity target red pulp myeloid cells for cancer immunotherapy
Source: Nat Nanotechnol. 2024 Jul 31;19(11):1735–44. doi: 10.1038/s41565-024-01727-w (PMC11567884; doi:10.1038/s41565-024-01727-w)
Supplement: Supplementary file 2 — Reporting Summary [file 41565_2024_1727_MOESM2_ESM.pdf]

nature portfolio

Corresponding author(s): Jan C.M. van Hest

Last updated by author(s): May 24, 2024

Reporting Summary

Nature Portfolio wishes to improve the reproducibility of the work that we publish. This form provides structure for consistency and transparency in reporting. For further information on Nature Portfolio policies, see our Editorial Policies and the Editorial Policy Checklist.

Statistics

For all statistical analyses, confirm that the following items are present in the figure legend, table legend, main text, or Methods section.

n/a	Confirmed	
☐	☒	The exact sample size (*n*) for each experimental group/condition, given as a discrete number and unit of measurement
☐	☒	A statement on whether measurements were taken from distinct samples or whether the same sample was measured repeatedly
☐	☒	The statistical test(s) used AND whether they are one- or two-sided<br>*Only common tests should be described solely by name; describe more complex techniques in the Methods section.*
☒	☐	A description of all covariates tested
☐	☒	A description of any assumptions or corrections, such as tests of normality and adjustment for multiple comparisons
☐	☒	A full description of the statistical parameters including central tendency (e.g. means) or other basic estimates (e.g. regression coefficient) AND variation (e.g. standard deviation) or associated estimates of uncertainty (e.g. confidence intervals)
☐	☒	For null hypothesis testing, the test statistic (e.g. *F*, *t*, *r*) with confidence intervals, effect sizes, degrees of freedom and *P* value noted<br>*Give P values as exact values whenever suitable.*
☒	☐	For Bayesian analysis, information on the choice of priors and Markov chain Monte Carlo settings
☒	☐	For hierarchical and complex designs, identification of the appropriate level for tests and full reporting of outcomes
☒	☐	Estimates of effect sizes (e.g. Cohen's *d*, Pearson's *r*), indicating how they were calculated

Our web collection on statistics for biologists contains articles on many of the points above.

Software and code

Policy information about availability of computer code

Data collection	No software code was used in order to collect data in this study.
Data analysis	OsiriX MD v12.0, FlowJo v10.9.0, Graphpad Prism v10, ImageJ v2.0.0, Excel Microsoft 365, Astra v7.3.2, MestReNova v11.0, Bruker Topspin v3.6.1, ZetaSizer Software v7.13, LabSolutions v5.73, Nanosight NTA 3.2, PerkinElmer SpectrumIR v10.6.1.942, Universal Analysis 2000 v4.5A, Bruker flexControl v3.4, Digital Micrograph v1.85.1535, Helios Software (Standard Biotools, CyTOF Software version v7.0), Tera-tomo 3D iterative reconstruction algorithm, Siemens proprietary e7tools with an Ordered Subset Expectation Maximization (OSEM) algorithm with Point Spread Function (PSF) correction.

For manuscripts utilizing custom algorithms or software that are central to the research but not yet described in published literature, software must be made available to editors and reviewers. We strongly encourage code deposition in a community repository (e.g. GitHub). See the Nature Portfolio guidelines for submitting code & software for further information.

Data

Policy information about availability of data

All manuscripts must include a data availability statement. This statement should provide the following information, where applicable:

- Accession codes, unique identifiers, or web links for publicly available datasets
- A description of any restrictions on data availability
- For clinical datasets or third party data, please ensure that the statement adheres to our policy

Raw data are available upon request. Other data are presented in the main text and supplementary information.

Human research participants

Policy information about studies involving human research participants and Sex and Gender in Research.

Reporting on sex and gender	N/A
Population characteristics	N/A
Recruitment	N/A
Ethics oversight	N/A

Note that full information on the approval of the study protocol must also be provided in the manuscript.

Field-specific reporting

Please select the one below that is the best fit for your research. If you are not sure, read the appropriate sections before making your selection.

☒ Life sciences ☐ Behavioural & social sciences ☐ Ecological, evolutionary & environmental sciences

For a reference copy of the document with all sections, see nature.com/documents/nr-reporting-summary-flat.pdf

Life sciences study design

All studies must disclose on these points even when the disclosure is negative.

Sample size	Based on our experience with the B16F10 murine melanoma model, we used group sizes of 10 animals for the tumor growth experiments. This group size provides enough power to detect statistically significant differences in growth rate and/or tumor size.<br>We used groups of similar sizes (i.e., 8 to 10 mice per group) to investigate differences in numbers of immune cells subsets in the spleen. These group sizes were also based on previous studies.
Data exclusions	We excluded blood samples from blood half-life analysis when they contained to little material (<0.001 g).
Replication	We have not attempted to replicate the in vivo β-glucan polymersomes therapeutic efficacy experiments (i.e., tumor growth measurements and flow cytometry analysis of the spleen). However, we did compare our control groups to those of previous experiments and found that we were able to successfully replicate our findings.
Randomization	Animals were allocated to different experimental designs on the basis of randomly chosen cage numbers.
Blinding	Data analysis and acquisition were performed by different individuals without knowledge of group allocation.

Reporting for specific materials, systems and methods

We require information from authors about some types of materials, experimental systems and methods used in many studies. Here, indicate whether each material, system or method listed is relevant to your study. If you are not sure if a list item applies to your research, read the appropriate section before selecting a response.

Materials & experimental systems

n/a	Involved in the study
	☒ Antibodies
	☒ Eukaryotic cell lines
☒	Palaeontology and archaeology
	☒ Animals and other organisms
☒	Clinical data
☒	Dual use research of concern

Methods

n/a	Involved in the study
☒	ChIP-seq
	☒ Flow cytometry
☒	MRI-based neuroimaging

Antibodies

Antibodies used

Flow cytometry:
anti-CD45 (clone 30-F11, BioLegend, 103138, 1:200),
anti-Ly6C (clone AL-21, BD Biosciences, 560592, 1:200),
anti-Ly6C (clone, HK1.4, BioLegend, 128006, 1:200),
anti-CD11b (clone M1/70, BioLegend, 101228, 1:200),
anti-CD11c (clone N418, BioLegend, 117310, 1:200),
anti-F4/80 (clone BM8, BioLegend, 123114, 1:100),
anti-CD90.2 (clone 53-2.1, BD PharMingen, 553006, 1:200),
anti-Ter119 (clone TER119, BD Biosciences, 51-09082J, 1:200),
anti-NK1.1 (clone PK136, eBioscience, 48-5941-82, 1:200),
anti-CD49b (clone DX5, eBioscience, 48-5971-82, 1:200),
anti-CD45R/B220 (clone RA3-6B2, eBioscience, 48-0452-82, 1:200),
anti-CD115 (clone AFS98, eBioscience, 17-1152-82, 1:200),
anti-Ly6G (clone 1A8, eBioscience, 48-9668-82, 1:100),
anti-Ly6G (clone 1A8, Biolegend, 127618, 1:100),
anti-CD19 (clone 1D3, BD PharMingen, 557399, 1:400).
anti-CD4 (clone RM4-4, BioLegend, 116016, 1:300)
anti-CD3 (clone 17A2, BioLegend, 100206, 1:300)
anti-CD8a (clone 53-6.7, BioLegend, 100708, 1:300)
anti-I-A/I-E (MHCII; clone M5/114.15.2, BioLegend, 107616, 1:250)
anti-Ly6G (clone 1A8, BioLegend, 127624, 1:250)
anti-CD115 (clone AFS98, BioLegend, 135517, 1:250)
anti-CD11b (clone M1/70, BioLegend, 101243, 1:200)
anti-PD-L1 (clone 10F.9G2, BioLegend, 124348, 1:100)
anti-CD45 (clone 30-F11, BioLegend, 103130, 1:100)

Mass cytometry:
anti-CD45 (clone 30-F11, BioLegend, 103102, 1:200)
anti-CD3 (clone 145-2C11, BioLegend, 100345, 1:50)
anti-CD11c (clone N418, BioLegend, 117302, 1:50)
anti-F4/80 (clone BM8, BioLegend, 123102, 1:200)
anti-Ly6C (clone HK1.4, BioLegend, 117302, 1:50)
anti-CD19 (clone 6D5, BioLegend, 115502, 1:50)
anti-Ly6G (clone 1A8, BioLegend, 127602, 1:200)
anti-Ter119 (clone TER-119, BioLegend, 116202, 1:200)
anti-CD200R3 (clone Ba160, BioLegend, 142302, 1:50)
anti-CD49b (clone DX5, BioLegend, 108902, 1:200)
anti-CD169 (clone 3D6.112, BioLegend, 142402, 1:100)
anti-CD115 (clone AFS98, BioLegend, 135521, 1:200)
anti-MARCO (clone EPR24317-33, Abcam, ab271060, 1:500)
anti-CD117 (clone 2B8, BioLegend, 105802, 1:883)
anti-NK1.1 (clone PK136, BioLegend, 108702, 1:50)
anti-CD172a (clone P84, BioLegend, 144002, 1:50)
anti-Sca-1 (clone D7, BioLegend, 108102, 1:200)

Validation

According to statements on the manufacturers websites, each antibody used in this study was validated for flow cytometry applications, and the following primary antibodies were validated for mass cytometry (CyTOF) applications: anti-CD45, anti-CD3 (clone 145-2C11), anti-CD11c, anti-Ly6C, anti-CD19, anti-CD115, anti-NK1.1, and anti-CD11b.
As stated on manufacturers/suppliers websites, the following primary antibodies have been validated for reactivity in mouse: anti-CD45, anti-Ly6C, anti-CD11c, anti-CD11b, anti-F4/80, anti-CD90.2, anti-Ter119, anti-NK1.1, anti-CD49b, anti-CD115, anti-Ly6G, anti-CD19, anti-CD4, anti-CD3 (clone 17A2), anti-CD8a, anti-I-A/I-E, anti-Ly6G, anti-PD-L1, anti-CD3 (clone 145-2C11), anti-CD19, anti-CD200R3, anti-CD169, anti-MARCO, anti-CD117, anti-CD172a, and anti-Sca-1.
As stated on manufacturers/suppliers websites, the following antibodies have been validated for reactivity in mouse and human: anti-CD45R/B220 and anti-CD11b.

Eukaryotic cell lines

Policy information about cell lines and Sex and Gender in Research

Cell line source(s)	B16-F10 (ATCC, CRL-6475 ™) cancer cell line provided by Dr. I.J. Fidler (MD Anderson Cancer Center, Houston, TX)
Authentication	B16-F10, morphology check by microscope showed mixture of spindle-shaped and epithelial-like cells. Cell line has not been authenticated after purchase.
Mycoplasma contamination	Cell lines were negative for mycoplasma contamination.
Commonly misidentified lines (See ICLAC register)	The study did not involve commonly misidentified cell lines.

Animals and other research organisms

Policy information about studies involving animals; ARRIVE guidelines recommended for reporting animal research, and Sex and Gender in Research

Laboratory animals	Female C57BL/6 mice (The Jackson Laboratory, JAX: 000664) and female B6;129S7-Rag1tm1Mom/J mice (The Jackson Laboratory, JAX:002096), all 8 weeks old. Two male adult cynomolgus monkeys (Macaca fascicularis) of 14 and 15 years old. All animals had free access to food and water. Mice were co-housed in climate-controlled rooms (ambient temperature and humidity) with 12-hour light/dark cycles. The mice were allowed to acclimate to the housing facility for at least 1 week before they were randomly assigned to experimental groups. Non-human primates were pair-housed, when possible, in climate-controlled conditions with 12 h light/dark cycles.
Wild animals	The study did not involve wild animals.
Reporting on sex	Female mice and male cynomolgus monkeys
Field-collected samples	The study did not involve samples collected from the field.
Ethics oversight	All animal experiments were performed in accordance with Icahn School of Medicine at Mount Sinai Institutional Animal Care and Use Committee (IACUC), VU University Medical Center and Radboud University Dierexperimentencomissie (DEC) guidelines as well as Dutch requirements and laws on animal experimentation.

Note that full information on the approval of the study protocol must also be provided in the manuscript.

Flow Cytometry

Plots

Confirm that:

- [x] The axis labels state the marker and fluorochrome used (e.g. CD4-FITC).
- [x] The axis scales are clearly visible. Include numbers along axes only for bottom left plot of group (a 'group' is an analysis of identical markers).
- [x] All plots are contour plots with outliers or pseudocolor plots.
- [x] A numerical value for number of cells or percentage (with statistics) is provided.

Methodology

Sample preparation	Flow cytometry analyses were done on cells from C57BL/6 mice. Mice were euthanized and perfused with cold PBS (20 mL). Femurs, lymph nodes, and spleens were collected and stored on ice. Bone marrow cells were flushed out of femurs and strained through a 70-µm strainer. Lymph nodes and spleens were fragmented and meshed through a 70-µm strainer. Bone marrow and spleen samples were incubated with lysis buffer and washed with FACS buffer (Dulbecco's PBS complemented with 1% FBS, 1 mM EDTA, 0.5% bovine serum albumin, and 0.1% NaN3).
Instrument	Data were acquired on a LSRFortessa (BD Bioscience) or a CytoFLEX LX (Beckmann Coulter).
Software	Data were analyzed using FlowJo v10.9.0 (Tree Star).
Cell population abundance	Cells were not sorted for this study.
Gating strategy	For all experiments, preliminary FSC-A/SSC-A gates were used to exclude debris. Subsequently, FSC-A/FSC-W and SSC-A/SSC-W gates were used to select singlets.<br>For the immune cell specificity study, in the following plots DAPI-, and CD45+ cells were selected. Examples of the subsequent plots are all shown in the or supplementary material.<br>Lymphocytes were identified as CD45+, CD11blow and Lin+.<br>Neutrophils were identified as CD45+, CD11bhigh and Lin+.

Myeloid cells were identified as CD45+, CD11b+ and Lin-/low.
Dendritic cells were identified as CD45+, CD11b+, Lin-/low, CD11chigh and F4/80low.
Macrophages were identified as CD45+, CD11b+, Lin-/low, CD11clow and F4/80high.
Monocytes were identified as CD45+, CD11b+, Lin-/low, CD11clow, F4/80low, and Ly6Chigh/low.
For the immunotherapy study, in the following plot Zombie Aqua- cells were selected. Examples of the subsequent plots are all shown in the supplementary material.
Lymphocytes were identified as CD11blow and Lin+ (CD19/CD90.2+) .
Myeloid cells were identified as CD11b+ and Lin-/low.
Neutrophils were identified as CD11b+, Lin-/low and, Ly6Ghigh and CD115low.
Monocytes were identified as CD11b+, Lin-/low, Ly6Glow and CD115high and Ly6Chigh/low.
For the marker expression and T cell suppression study, in the following plots CD45+ and ViaKrome808- cells were selected. Examples of the subsequent plots are all shown in the supplementary material.
T cells were identified as CD45+, CD11blow and CD3+.
CD4+ T cells were identified as CD45+, CD11blow, CD3+ and CD4+.
CD8+ T cells were identified as CD45+, CD11blow, CD3+ and CD8a+.
Myeloid cells were identified as CD45+, CD11b+ and CD3-/low.
Neutrophils were identified as CD11b+, CD3-/low and, Ly6Ghigh and CD115low.
Monocytes were identified as CD11b+, CD3-/low, Ly6Glow and CD115high.

☒ Tick this box to confirm that a figure exemplifying the gating strategy is provided in the Supplementary Information.
